# Adapting to change: Exploring the consequences of climate‐induced host plant shifts in two specialist Lepidoptera species

**DOI:** 10.1002/ece3.11596

**Published:** 2024-06-25

**Authors:** Baptiste Bovay, Patrice Descombes, Yannick Chittaro, Gaëtan Glauser, Hanna Nomoto, Sergio Rasmann

**Affiliations:** ^1^ Faculty of Science, Institute of Biology University of Neuchâtel Neuchatel Switzerland; ^2^ Département de Botanique Muséum cantonal des sciences naturelles Lausanne Switzerland; ^3^ Info Fauna Neuchatel Switzerland; ^4^ Faculty of Science, Neuchâtel Platform of Analytical Chemistry University of Neuchâtel Neuchatel Switzerland

**Keywords:** elevation gradients, global warming, host plant shifts, life‐history traits, plant defences, plant–herbivore interactions, secondary metabolites, transplant experiment

## Abstract

Asynchronous migration of insect herbivores and their host plants towards higher elevations following climate warming is expected to generate novel plant–insect interactions. While the disassociation of specialised interactions can challenge species' persistence, consequences for specialised low‐elevation insect herbivores encountering novel high‐elevation plants under climate change remain largely unknown. To explore the ability of two low‐elevation Lepidoptera species, *Melitaea celadussa* and *Zygaena filipendulae*, to undergo shifts from low‐ to high‐elevation host plants, we combined a translocation experiment performed at two elevations in the Swiss Alps with experiments conducted under controlled conditions. Specifically, we exposed *M*. *celadussa* and *Z*. *filipendulae* to current low‐ and congeneric high‐elevation host plants, to test how shifts in host plant use impact oviposition probability, number of eggs clutches laid, caterpillar feeding preference and growth, pupation rate and wing size. While our study shows that both *M*. *celadussa* and *Z*. *filipendulae* can oviposit and feed on novel high‐elevation host plants, we reveal strong preferences towards ovipositing and feeding on current low‐elevation host plants. In addition, shifts from current low‐ to novel high‐elevation host plants reduced pupation rates as well as wing size for *M*. *celadussa*, while caterpillar growth was unaffected by host plant identity for both species. Our study suggests that populations of *M*. *celadussa* and *Z*. *filipendulae* have the ability to undergo host plant shifts under climate change. However, these shifts may impact the ability of populations to respond to rapid climate change by altering developmental processes and morphology. Our study highlights the importance of considering altered biotic interactions when predicting consequences for natural populations facing novel abiotic and biotic environments.

## Graphical abstract

Our study is the first to our knowledge exploring consequences for specialised low‐elevation insect herbivores facing novel, congeneric, high‐elevation plants. Importantly, our study highlights that considering altered biotic interactions, such as those between specialised insects and their host plants, is crucial to predict how species will respond to climate change.

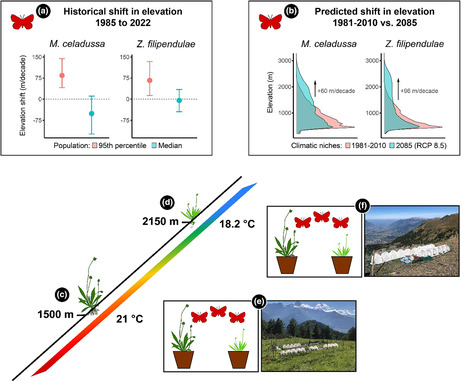

## 1 INTRODUCTION

Understanding how species respond to alterations in their abiotic and biotic environment is crucial to predict the ability of natural populations facing climate change to persist (IPBES, 2019). Apart from coping with climate change in situ, species can track their optimal temperatures by migrating towards higher latitudes (Forister et al., 2010; Parmesan & Yohe, 2003; Walther et al., 2002) and altitudes (Chen et al., 2009; Parolo & Rossi, 2008; Pauli et al., 2012). However, the capacity and rate of migration towards climatically suitable habitats often differ between taxonomic groups (Urban et al., 2012). For example, limited dispersal capacity can cause migration rates of plants to lag behind changes in temperatures (Alexander et al., 2018; Ash et al., 2017; Corlett & Westcott, 2013), while ectothermic insects are more likely to keep up with climate change via rapid migration (Rödder et al., 2021; Vitasse et al., 2021). Asynchronous migration of plants and insects following climate change (Kerner et al., 2023; Vitasse et al., 2021) is thus expected to generate novel plant–insect interactions involving species whose ranges are currently non‐overlapping (HilleRisLambers et al., 2013; Urban et al., 2012). Shifts in biotic interactions, such as those between plants and herbivores, pollinators and/or competitors, following altered abiotic conditions under climate change are described as indirect effects of climate change. Although these indirect effects can play crucial roles in dictating species' responses to climate change (Alexander et al., 2015; Descombes, Kergunteuil, et al., 2020; Gilman et al., 2010), consequences of altered plant–insect interactions following asynchronous range shifts on species' performance and development remain understudied.

To understand how species will respond to climate change, it is necessary to expose natural populations to abiotic and biotic conditions they are expected to face in the future. Mountains serve as unique study systems allowing for simulations of realistic future scenarios of both warmer climates and novel biotic interactions (Nooten & Hughes, 2017; Tito et al., 2020). Natural temperature gradients of mountains allow for simulations of predicted climate warming by translocating species downhill (Tito et al., 2020). Moreover, elevation gradients can be used for addressing the effect of novel plant–insect interactions formed because of asynchronous upslope migration under climate change. Previous studies simulating future scenarios of climate warming and altered interactions between plants and insects by transplanting plant and/or insect communities across elevation gradients have revealed that the establishment of low‐elevation insects at high elevation plays an important role in shaping plant community responses to climate change (Descombes, Kergunteuil, et al., 2020; Descombes, Pitteloud, et al., 2020; Richman et al., 2020). For example, Descombes, Pitteloud, et al. (2020) showed that the introduction of low‐elevation generalist insect herbivores at high elevation can alter biomass production, diversity and chemical properties of alpine plant communities. While the consequences of altered plant–insect interactions mainly have been explored by estimating responses in plant communities, insect herbivores are also expected to face challenges when encountering novel plant communities. Specifically, the absence of current host plants can threaten the persistence of insect herbivores by limiting their capacity to establish at higher elevations, unless they are able to shift their diet and consume new plant species (Hanspach et al., 2014; Merrill et al., 2008).

Host plant shifts can impact insect herbivores by altering vital rates associated with reproduction and growth, where interactions with a novel host plant may reduce oviposition success, offspring survival and size (Braschler & Hill, 2007; Näsvall et al., 2021; Parry & Goyer, 2004; Pelini et al., 2009). While generalist insect herbivores are expected to successfully establish novel plant communities (Berg et al., 2010; Gilman et al., 2010; Rödder et al., 2021), specialists might be less likely to do so (Andrew & Hughes, 2004; Warren et al., 2001; Yamamoto & Uchida, 2018). Specialised plant‐insect herbivore interactions are shaped by co‐evolutionary arms races (Becerra et al., 2009; Ehrlich & Raven, 1964), mediated by the reciprocal evolution of chemical defences produced by host plants and detoxification/sequestration mechanisms by insect herbivores. Hence, long periods of co‐evolution have optimised performance on one or a limited number of plant species, which may constrain the performance of other plant species (Futuyma & Moreno, 1988; Pelini et al., 2009). In this regard, successful establishment of novel species requires that adult can recognise the novel plants, mostly via chemical cues (Honda, 1995; Nishida, 2014) and that larvae can survive and grow on leaves containing (slightly) different secondary metabolites produced by these plants (Jeschke et al., 2017; Pelini et al., 2009, 2010).

Accordingly, theory suggests that specialised insect herbivores could recognise, survive and develop on other plant species if their phenotypes largely match those of the current host plants (Gassmann et al., 2006). It is thus expected that specialised low‐elevation insect herbivores encountering alpine plant communities will choose to oviposit and feed on species closely related to their current low‐elevation host plants (Moir et al., 2014), as these not only are likely to be morphologically but also chemically similar (Fahey et al., 2001; Farrell & Mitter, 1998; Griffin & Lin, 2000). However, although they might be chemically similar, even small variations in chemistry can generate widespread impacts on the insect herbivores (Glassmire et al., 2016; Harrison et al., 2016), which may impact the likelihood of successful colonisation of the novel host plant. Therefore, to estimate the consequences for specialised low‐elevation insect herbivores encountering high‐elevation plants due to climate warming, it is essential to study both adult responses and the ability of larvae to adapt to novel host plants.

We here explored how shifts from current low‐ to novel high‐elevation host plants impact the ability of two specialist Lepidoptera species, *Melitaea celadussa* (Nymphalidae) and *Zygaena filipendulae* (Zygaenidae), to establish at higher elevations. Specifically, we asked: (1) Can *M*. *celadussa* and *Z*. *filipendulae* oviposit and feed on novel high‐elevation host plants? (2) How do shifts from current low‐ to novel high‐elevation host plants impact caterpillar growth, pupation rate and wing size? (3) Are high‐ and low‐elevation congeneric plants phenotypically and chemically different? Overall, we hypothesised *M*. *celadussa* and *Z*. *filipendulae* prefer to oviposit and feed on current low‐elevation host plants, while we expected reduced chemical defence in high‐elevation plants to contribute to enhanced performance of caterpillars feeding on high‐ compared to low‐elevation plants.

To address our questions, we combined a field reciprocal transplant experiment established at two elevations with experiments performed under laboratory conditions. By exposing *M*. *celadussa* and *Z*. *filipendulae* to current low‐ and novel high‐elevation host plants at both high‐ and low‐elevation under field conditions, we explored how shifts in host plant identity under different climates impact oviposition probability and success (estimated as the number of egg clutches) of *M*. *celadussa* and *Z*. *filipendulae*. As for other taxonomic groups (Dahlke et al., 2020; Doak & Morris, 2010), responses to climate change in Lepidoptera can vary between life stages (Radchuk et al., 2013). Therefore, apart from oviposition, we explored how shifts in host plant identity impact caterpillar preference and performance (estimated by growth; Knolhoff & Heckel, 2014), pupation rate and wing size, by conducting additional experiments exposing *M*. *celadussa* and *Z*. *filipendulae* to current low‐ and novel high‐elevation host plants under controlled conditions. Finally, we performed targeted metabolomic analyses on plants to explore whether responses to shifts in host plant identity were associated with differences in concentrations of chemical defence compounds.

## 2 MATERIALS AND METHODS

### 2.1 Study system

As focal species, we selected two Lepidoptera species abundant and non‐threatened in Switzerland (Wermeille et al., 2014); the Southern heath fritillary *Melitaea celadussa* Fruhstorfer, 1910, and six‐spot burnet *Zygaena filipendulae* (Linnaeus, 1758). *Melitaea celadussa* is a butterfly ovipositing egg clutches of c. 50–100 eggs on species belonging to the Plantaginaceae family, mainly on *Plantago lanceolata* L. While *P*. *lanceolata* is considered to be the main hostplant, *M*. *celadussa* has also been recorded to oviposit on a few species belonging to the families Orobanchaceae and Asteraceae, but, to our knowledge, never on alpine *Plantago* species (Clarke, 2022; LSPN, 1987). *Melitaea celadussa* populations are found between 400 and 1400 m within the study region (Figure S1a). *Zygaena filipendulae* is a diurnal burnet moth exclusively laying egg clutches of c. 50–100 eggs on plants belonging to the genus *Lotus* (particularly on *Lotus corniculatus* L. in the Alps). Moreover, caterpillars of *Z*. *filipendulae* have been observed to feed on other Fabaceae species, but again, to our knowledge, never observed feeding on alpine *Lotus* species (Guenin, 2023; LSPN, 1999; Paolucci, 2013). The elevational distribution of *Z*. *filipendulae* in the study region ranges mainly between 400 and 1600 m (85% of the observations are below 1560 m, Figure S1b).

**FIGURE 1:**
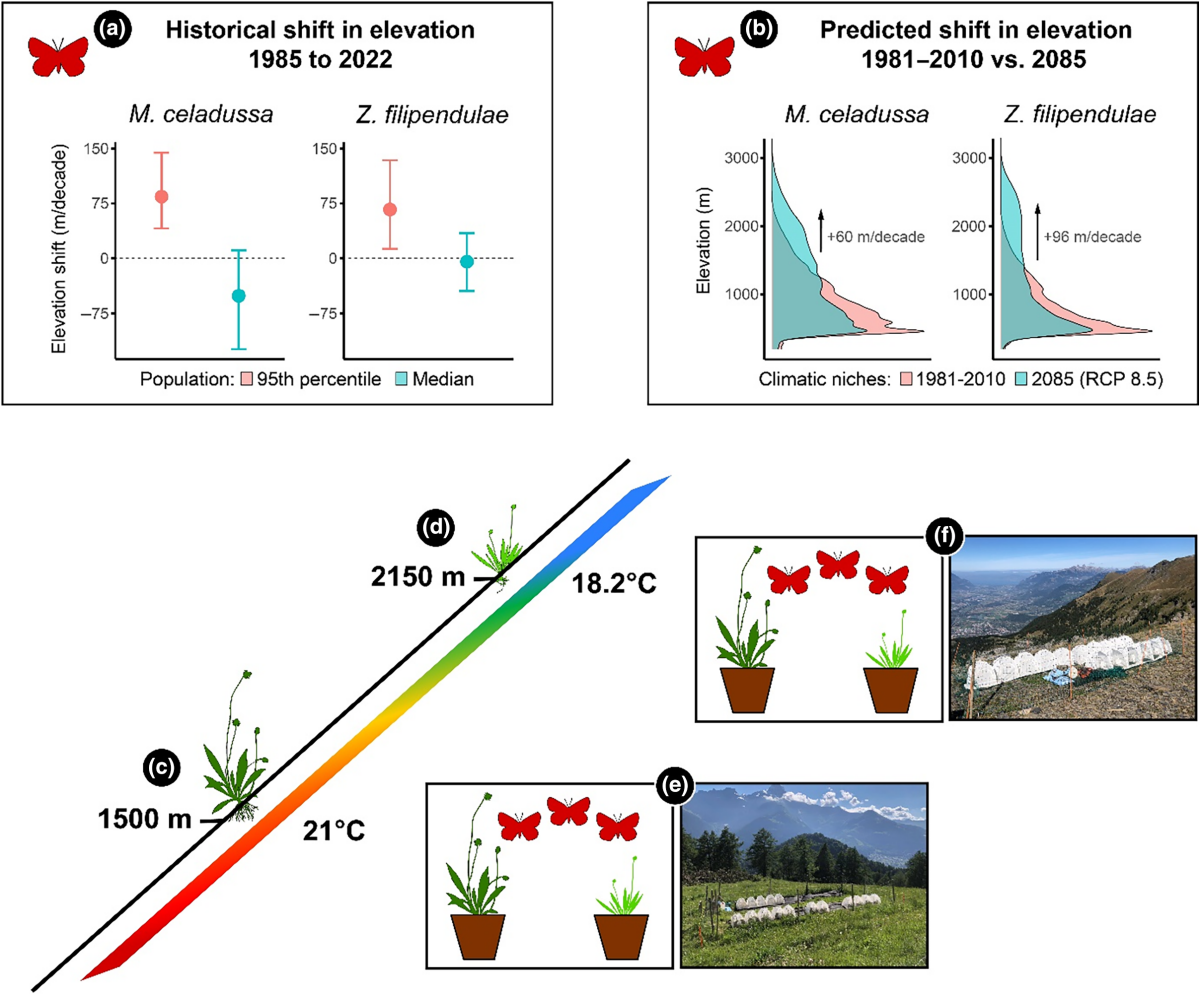
Migration rates of *Melitaea celadussa* and *Zygaena filipendulae* (a and b) and the experimental design for field experiment (c–f). Both *M*. *celadussa* and *Z*. *filipendulae* are currently migrating towards higher elevations and are expected to migrate at a similar rate over the coming decades. (a) Illustrates the current shift in elevation of our focal Lepidoptera species in Switzerland (*y*‐axis) of both the 95th percentile and the median of the elevation distributions (*x*‐axis). Points illustrate the average shift in elevation for 37 years (1985–2022) in Switzerland, and error bars indicate 95% confidence intervals. (b) Illustrates the shift in elevation of the climatic niches of the Lepidoptera by comparing their current (1981–2010) and future (RCP 8.5, 2085, i.e. average of 2070–2100) climatic niches. The plots represent the density of suitable environments in Switzerland (*x*‐axis) according to the elevation (*y*‐axis). To simulate a realistic migration range, two common gardens were set up in the Western Swiss Alps at 1500 m (c) and 2150 m (d), thus representing an elevation shift of c. 650 m. Each common garden included cages containing two females and one male (e and f) of either *M*. *celadussa* or *Z*. *filipendulae* originating from low elevation together with one congeneric pair of *Plantago* spp. (*P*. *lanceolata*–*P*. *atrata*; *n* = 9 cages/site) or *Lotus* ssp. (*L*. *corniculatus*–*L*. *alpinus*; *n* = 15 cages/site) respectively. By quantifying oviposition probability and success of Lepidoptera exposed to current low‐ and novel high‐elevation host plants under warmer (1500 m) and cooler (2150 m) climates, we investigated the effects of host plant identity and climate on oviposition.

For both species, we estimated their potential distribution following realistic scenarios of climate change (see Methods S1 section). Between 1985 and 2022, leading edges of elevation distributions (95th percentile, estimated following the methods used in Vitasse et al., 2021) have shifted towards higher elevations at a rate of 84.2 and 66.7 m/decade, for *M*. *celadussa* and *Z*. *filipendulae*, respectively (Figure 1a). In addition, predictions obtained from species distribution models (SDMs) using current occurrences of both Lepidoptera species in Switzerland suggest that leading edges of species' climatic niches will shift towards higher elevations by the end of the century following climate change. Specifically, an increase in global average temperatures by 1.1–2.6°C (Representative Concentration Pathways 4.5), as predicted by the end of the century (IPCC, 2014, 2023), is expected to shift leading edges upwards by 227 and 494 m for *M*. *celadussa* and *Z*. *filipendulae*, respectively (Figures S2 and S3). Under climate change scenario Representative Concentration Pathways 8.5, assuming that global average temperatures rise by 2.6–4.8°C by the end of the century (IPCC, 2014, 2023), leading edges are predicted to shift by 544 and 868 m for *M*. *celadussa* and *Z*. *filipendulae*, respectively (Figure 1b and Figures S2 and S3).

The lowland plant species *P*. *lanceolata* and *L*. *corniculatu*s were selected to represent current low‐elevation host plants for *M*. *celadussa* and *Z*. *filipendulae*, respectively (Guenin, 2023; LSPN, 1987, 1999). As congeneric high‐elevation species, we selected *Plantago atrata* Hoppe and *Lotus alpinus* (DC.) Ramond for *M*. *celadussa* and *Z*. *filipendulae*, respectively, as they are the closest, most abundant, high‐elevation relatives to the selected low‐elevation host plants. To date, *M*. *celadussa* and *Z*. *filipendulae* have never been observed feeding or ovipositing on these high‐elevation plants. Both *P*. *lanceolata* and *P*. *atrata* are known to produce iridoid glycosides (IGs), acting not only as feeding deterrent compounds for several generalist herbivores (Biere et al., 2004; Rønsted et al., 2002) but also as feeding and oviposition stimulants for more specialist herbivores (Bowers, 1983; Nieminen et al., 2003; Pereyra & Bowers, 1988; Reudler Talsma et al., 2008). *Lotus corniculatus* and *L*. *alpinus* produce cyanogenic glycosides (CNGs), known not only to deter generalist herbivores via activation by β‐glucosidase enzymes but also to protect specialists via sequestration (Zagrobelny et al., 2007). For more information on Lepidoptera and plant species used in this study, see Figures S1–S4 and Table S1. For sample sizes as well as the caterpillar instars used for experiments performed in field and under controlled conditions (see below), see Table S2.

### 2.2 Oviposition experiment

To explore how shifts in host plant identity and different climatic conditions influence the probability of oviposition and the number of egg clutches laid by *M*. *celadussa* and *Z*. *filipendulae*, we established two common garden experiments at two different elevations in the Swiss Alps in June 2022 (Figure 1c–f). The experiment included two sites, one situated at 1500 and one at 2150 m, selected specifically to ensure similarities in sun exposure, slope, and orientation (Table S3). Average temperatures throughout the experiment (June–July) corresponded to 21 and 18.2°C for the low‐ and high‐elevation sites, respectively (Table S3, Figure S5).

Individuals of *P*. *lanceolata* (*n* = 20) and *L*. *corniculatus* (*n* = 30) were excavated in the surroundings of the experimental site situated at 1500 m, while *P*. *atrata* (*n* = 20) and *L*. *alpinus* (*n* = 30) were excavated near the 2150 m site (Table S4). Selected individuals grew at least 1 m apart from each other and were carefully excavated to prevent damage to below‐ground structures. Only individuals with no or minimal herbivory damage were selected. Individuals were then planted into pots (20 cm diameter) with standard potting soil (Terreau Suisse, Ricoter, Aarberg, Switzerland), and reciprocally translocated to 1500 and 2150 m (Figure 1). High‐elevation plants translocated to 1500 m and thus experienced an increase in temperature of 2.8°C on average, simulating climate warming. Although morphologically similar (Lauber et al., 2018), high‐elevation individuals produced shorter stalks, but more leaves and stalks (see Methods S1 for trait measurements, Figure S6; Table S5). In addition, the leaf C:N ratio was higher for individuals of *P*. *atrata* compared to *P*. *lanceolata* (Figure S7; Table S6).

For the oviposition choice experiment, one individual of the low‐elevation plant species was placed together with an individual of the congeneric high‐elevation species in cages (*n* = 2 individuals/cage) designed for butterfly rearing (50 × 50 × 70 cm, BugDorm‐2S120, MegaView Science Co., Ltd, Taichung, Taiwan), generating a total of *n* = 11 and *n* = 15 congeneric pairs for *Plantago* spp. and *Lotus* spp., respectively, at low elevation and *n* = 9 and *n* = 15 congeneric pairs for *Plantago* spp. and *Lotus* spp., respectively, at high elevation. The bottoms of the cages were covered by a nylon mesh to prevent underground disturbance from local arthropod communities. Plants were allowed to acclimatise after translocation for 7 days before *M*. *celadussa* and *Z*. *filipendulae* adults were added into cages.

*Melitaea celadussa* and *Z*. *filipendulae* were captured at multiple sites near the study area (between c. 400 and 1460 m; Table S5). In an optimal situation, Lepidoptera individuals would have been collected from multiple low‐elevation sites at the same elevation, to avoid potential “home‐elevation” effects. However, this was not possible in this study, as the number of populations of *M*. *celadussa* and *Z*. *filipendulae* in the study area is limited. Hence, to avoid imposing major effects on population size and to maximise the genetic variation, we sampled individuals from multiple populations at different elevations. Importantly, the aim of this study was to expose Lepidoptera species to future high‐elevation host plants, with whom they never interacted before. Although absolute elevations from which Lepidoptera included in the field experiment originated varied, selected high‐elevation plant species were absent from all collection sites as they started to appear at an elevation of c. 1750 m in the region where Lepidoptera were collected. Therefore, high‐elevation plant species represent novel hosts for all Lepidoptera individuals included in the experiment. After their capture, Lepidoptera were stored in cylindrical plastic containers and sexed (see Methods S1). Two females and one male of *M*. *celadussa* (total *n* = 60 individuals) and *Z*. *filipendulae* (total *n* = 90 individuals) were placed in each cage prepared as described above within 24 h following collection. With this set‐up, we simulated scenarios where *M*. *celadussa* and *Z*. *filipendulae* can encounter current low‐ and potential novel high‐elevation host plants simultaneously under warmer (1500 m) and cooler (2150 m) climates. Three days after releasing *M*. *celadussa* and Z. *filipendulae* individuals in the cages, the presence/absence and the number of egg clutches laid on each plant were recorded. Surviving *M*. *celadussa* and *Z*. *filipendulae* individuals were released at the sites from which they were captured. The field experiment was performed during June–July since not all Lepidoptera could be captured simultaneously for filling all the replicates per treatment. At the end of the experiment, all plants (including egg clutches) were brought to the University of Neuchâtel and used for experiments performed under controlled conditions and for chemical analyses of plants (see below).

To determine whether host plant identity and elevation influenced the oviposition preference of *M*. *celadussa* and *Z*. *filipendulae*, we fitted a generalised linear model (GLM) with binomial family distribution for each Lepidoptera species separately, where the probability of laying eggs was implemented as the response variable and the interactive effect between plant species and the elevation (high or low elevation) as explanatory variables. To test whether host plant identity and elevation influence oviposition success, we fitted GLMs with Poisson distribution for each Lepidoptera species separately, where the number of egg clutches was implemented as the response variable and elevation, plant species and the interactive effect between plant species and elevation as explanatory variables. Effects of host plant identity and elevation on oviposition preference and success were estimated by performing Chi^2^ tests of fitted models using analyses of variance (ANOVA). Finally, as the focus of these analyses was imago preferences, cages where Lepidoptera did not oviposit were excluded from the statistical analyses (final sample size *n* = 5 for *Z*. *filipendulae* in each elevation and *n* = 10 and *n* = 6 for *M*. *celadussa* at low and high elevation, respectively).

### 2.3 Effects of hostplant identity on the preference and performance of caterpillars

To investigate whether host plant identity impacts the performance and preference of caterpillars, pupation rate and adult wing size, eggs obtained from the field experiment were used to rear caterpillars under optimal climatic conditions (16 h of daylight, 27 and 22°C of average day and night temperature, respectively).

#### 2.3.1 Caterpillar preference

To explore whether caterpillars prefer low‐ or high‐elevation plant species, we placed caterpillars, being between the third and the sixth instars, in the centre of a Petri dish (9 cm × 1.4 cm) containing either whole leaves of both *Lotus* spp. or 20 × 5 mm leaf rectangles of both *Plantago* spp. High‐elevation plants were collected at around 2150 m, while low‐elevation plants were collected at 500 m (Table S4). Plants were then left to acclimatise for 7 days under climate‐controlled conditions before leaf sampling. Leaf area was estimated using ImageJ (Schneider et al., 2012) before being placed equidistant from the centre of a Petri dish. After 5 or 3 h, depending on differences in feeding rate between *M*. *celadussa* and *Z*. *filipendulae*, the leaf area was estimated again, and the consumed leaf area was calculated by subtracting the initial from the final leaf area. To avoid biases due to potential preference towards the plant species on which caterpillars initially hatched and fed (before reaching third instar; Knolhoff & Heckel, 2014), preference was tested for *M*. *celadussa* caterpillars initially reared on either *P*. *lanceolata* (*n* = 19) or *P*. *atrata* (*n* = 20). Similarly, for *Z*. *filipendulae*, the experiment was performed on caterpillars initially reared on either *L*. *corniculatus* (*n* = 20) or *L*. *alpinus* (*n* = 20).

To estimate the effects of host plant identity on caterpillar preference, we accounted for the potential effects of initial leaf size on the leaf area consumed by caterpillars of *M*. *celadussa* and *Z*. *filipendulae* by extracting residuals of a linear model including log‐transformed leaf area consumed as response variable and log‐transformed initial leaf size as explanatory variable. Log transformations of the leaf area consumed and initial leaf area were performed to obtain normally distributed residuals. To test whether host plant identity impacts the preference of caterpillars, we fitted linear mixed effects (LME) models for each Lepidoptera species separately, where residuals of linear models described above were implemented as the response variable, host plant identity as the explanatory variable and host plant identity on which caterpillars had fed on before the start of the preference experiment as a random factor. Effects of host plant identity on caterpillar preference were estimated by performing Chi^2^ tests of fitted models using ANOVA.

#### 2.3.2 Caterpillar performance

To test whether host plant identity and translocation of plants to different elevations (i.e. different climates) impact caterpillar performance, we exposed caterpillars (second or third instar) to low‐ and high‐elevation host plants that were used at both elevations in the field experiment (Figure 1c–f). After estimating initial weight, one caterpillar was placed in the centre of a Petri dish (9 × 1.4 cm) containing one leaf for *Plantago* spp., or one stem with several leaves for *Lotus* spp. For *M*. *celadussa*, caterpillars were placed into separate Petri dishes containing leaves originating from *P*. *lanceolata* individuals translocated at the experimental site situated at low (*n* = 42) and high elevation (*n* = 43), as well as *P*. *atrata* individuals, translocated to the experimental site situated at low (*n* = 46) and high elevation (*n* = 36). Similarly, for *Z*. *filipendulae*, caterpillars were exposed to leaves originating from *L*. *corniculatus* individuals translocated to the experimental site situated at low (*n* = 30) and high elevation (*n* = 27) as well as *L*. *alpinus* individuals translocated to the experimental site situated at low (*n* = 20) or high elevation (*n* = 30). Cut parts of leaves were wrapped in moist cotton and aluminium to keep leaves fresh (Descombes et al., 2017). After 4 days, caterpillars were weighed and the growth rate was calculated by subtracting the final from the initial weight. Differences in weight were divided by the number of days to estimate growth rate (mg/day).

To explore whether host plant identity and translocation treatments impact the performance of caterpillars, we fitted linear models for *M*. *celadussa* and *Z*. *filipendulae* separately, where growth rate (mg/day) was implemented as the response variable and the elevation where host plants were acclimatised, host plant identity and the interactive effect between host plant identity and elevation as explanatory variables. To obtain normally distributed residuals, growth rate was log‐ and square‐root transformed for *M*. *celadussa* and *Z*. *filipendulae* respectively. Additionally, negative or null growth rates were removed prior to the analyses, as they indicate that caterpillars either did not feed or died. Effects of host plant identity and experimental site to which plants had been translocated on caterpillar performance were estimated by performing *F*‐tests of fitted models using ANOVA. To explore pair‐wise differences in growth rate between host plants and translocation treatments, we performed post hoc tests (pairwise comparisons with Tukey adjustment).

### 2.4 Effects of hostplant identity on the pupation rate and wing size of *M*. *celadussa*

For *M*. *celadussa*, we further investigated whether host plant identity impacts pupation rate and adult wing size by performing additional experiments under controlled conditions. These experiments could not be performed for *Z*. *filipendulae*, as all caterpillars from this species entered diapause, and thus released to the site of origin of their parent or died. To estimate the effect of host plant identity on pupation rate and wing size for *M*. *celadussa*, we placed *n* = 10 and 9 replicates of three second instar caterpillars on living plants growing in butterfly‐rearing cages on *P*. *lanceolata* and *P*. *atrata* respectively. All caterpillars used in this experiment hatched on the same day. As for the experiment performed to explore caterpillar preference, *P*. *atrata* individuals were collected around 2150 m, while *P*. *lanceolata* individuals were collected at 500 m (Table S4). Plants were potted and left to acclimatise for 7 days under controlled conditions in the greenhouse before caterpillars were placed on plants. Caterpillars were placed on the same plant species on which they hatched.

#### 2.4.1 Pupation rate

To investigate whether host plant identity impacts the pupation rate for *M*. *celadussa*, we recorded whether caterpillars (total *n* = 30 and 27 for *P*. *lanceolata* and *P*. *atrata*, respectively) entered pupation or diapause. For details on how pupation/diapause was determined, see Methods S1. To test whether the identity of the host plants on which *M*. *celadussa* caterpillars fed impacted their probability of entering pupation (pupation rate), we fitted GLMs with a binomial family distribution with recordings of pupation (pupation = 1, diapause = 0) as the response variable and host plant identity as an explanatory variable. Effects of host plant identity on pupation rate were estimated by performing Chi^2^ tests of fitted models using ANOVA.

#### 2.4.2 Wing size

To test whether host plant identity impacts wing size, we estimated the wing area of all individuals reaching adult stage (*n* = 17 and 11 individuals reared on *P*. *lanceolata* and *P*. *atrata*, respectively) of *M*. *celadussa*. Dead adults were dried at room temperature for c. 1 month before wings were removed. Wing area was estimated using ImageJ (Schneider et al., 2012). Perimeters of torn wings were corrected (*n* = 8) and when large proportions of a wing were damaged (*n* = 3), measurements of the opposite wing were used to estimate total wing area. To test whether the wing size of individuals reaching the adult stage after pupation is affected by host plant identity, we fitted linear models with square‐root‐transformed wing area as the response variable and host plant identity as an explanatory variable. Square‐root transformation of wing size was performed to obtain normally distributed residuals. Effects of host plant identity on wing area were estimated by performing *F*‐tests of fitted models using ANOVA.

### 2.5 Chemical analyses and data processing

To test whether the production of secondary metabolites related to anti‐herbivore defences varies according to host plant identity and climatic conditions, we performed targeted secondary metabolites analyses on a total of *n* = 59 randomly selected plant individuals included in the field experiment (*n* = 7, 7, 7, 8 individuals growing at experimental sites situated at low elevation and *n* = 8, 8, 6, 8 individuals growing at experimental sites situated at high elevation for *P*. *lanceolata*, *P*. *atrata*, *L*. *corniculatus* and *L*. *alpinus*, respectively). For *Plantago* spp., we analysed two iridoid glycosides (IGs), catapol and aucubin (Rønsted et al., 2000), while two cyanogenic glycosides (CNGs), linamarin and lotaustralin (Sun et al., 2018), were analysed for *Lotus* spp. For the detailed explanation of the chemical analytical procedures employed, see Methods S1.

To test whether the production of chemical defence compounds was impacted by host plant identity and translocation treatments, we fitted separate linear models for each compound (catapol, aucubin, linamarin and lotaustralin), where log‐transformed compound concentrations were implemented as response variables and the host plant identity, the experimental site to which plants were translocated, the interactive effect between host plant identity and experimental site to which plants were translocated as explanatory variables. Log transformations of compound concentrations were performed to obtain normally distributed residuals. Effects of host plant identity on secondary metabolites concentration were estimated by performing *F*‐tests of fitted models using ANOVA.

All statistical analyses were performed using R v.4.1.2 (R Core Team, 2021) and RStudio (RStudio Team, 2021). GLMs were fitted using the package *Stats* (R Core Team, 2021) while LMEs were fitted using the package *lmerTest* (Kuznetsova et al., 2017). Models were evaluated using the packages *DHARMa* (Hartig, 2022) and *Stats* (R Core Team, 2021). ANOVAs were performed using the package *car* (Fox & Weisberg, 2019). Pair‐wise differences in caterpillar growth rate between host plants and translocation treatments were estimated using the package *emmeans* (Lenth, 2022). Figures were produced using *ggplot2* package (Wickham, 2016).

## 3 RESULTS

### 3.1 Oviposition preference and success

**FIGURE 2:**
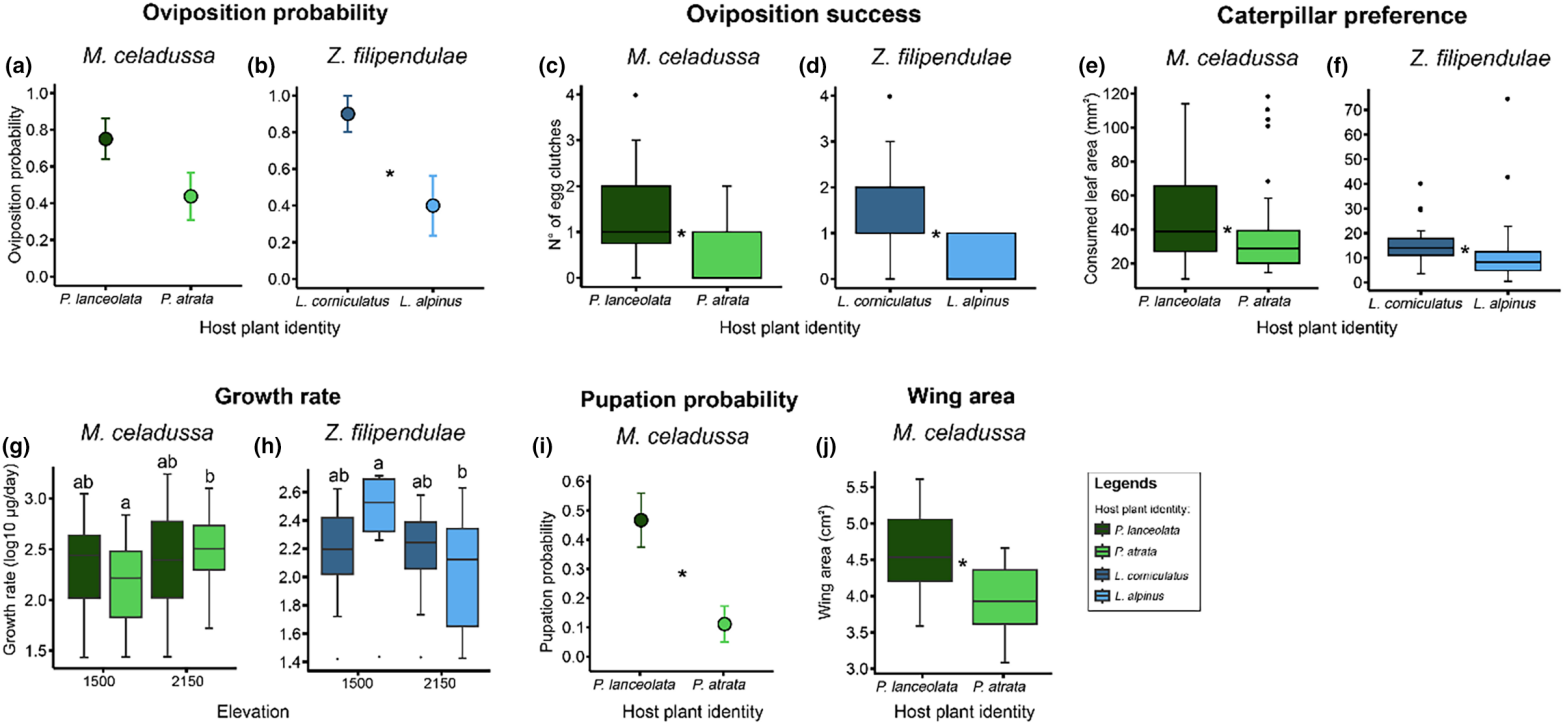
Oviposition probability (a and b), oviposition success (c and d), caterpillar preference (e and f), caterpillar growth rate (g and h), pupation rate (i) and wing area (j) across host plant identities (a–j) and elevation (g and h) for *Melitaea celadussa* and *Zygaena filipendulae*. Note that for a–d, effects of elevation are not illustrated as experimental site (1500/2150 m) neither impacted oviposition probability (a and b) nor the number of egg clutches (c and d; Table 1). For effects of elevation, see Figure S8 and Table 1. Colours indicate host plant identities where *Plantago lanceolata* and *Plantago atrata* are illustrated in dark and light green, respectively, and *Lotus corniculatus* and *Lotus alpinus* in dark and light blue respectively. For boxplots (c–h, j), bold, horizontal lines show medians, error bars illustrate distributions of the first to fourth quantile, and points represent outliers. For (a and b, i), points indicate means and error bars illustrate ±1 standard error. Asterisks (a–f, i and j) and letters (g and h) indicate significant differences (*p* < .05), which were calculated using *F*‐test (g and h, j) or Chi^2^ test (a–f, i). For details on significant effects of host plant identity and elevation on oviposition probability, number of egg clutches, consumed leaf area, caterpillar growth rate, pupation rate and wing area, see Table 1.

Host plant identity had no impact on oviposition probability for *M*. *celadussa* (Figure 2a; Table 1), while the probability of *Z*. *filipendulae* to oviposit on its current low‐elevation host plant, *L*. *corniculatus*, was 2.25× higher on average compared to *L*. *alpinus* (Figure 2b; Table 1). 2.3× and 4× more egg clutches were laid on current low‐ compared to congeneric high‐elevation host plants for *M*. *celadussa* (Figure 2c; Table 1) and *Z*. *filipendulae* (Figure 2d; Table 1) respectively. In contrast, elevation neither impacted oviposition probability nor the number of egg clutches laid (Table 1).

**TABLE 1:** Effects of host plant identity and translocation treatment (elevation) on oviposition probability, number of egg clutches, caterpillar preference and caterpillar growth rate for *Melitaea celadussa* and *Zygaena filipendulae* as well as pupation rate and wing size for *M*. *celadussa*.

Response variable	Species	Term	*χ* ^2^/*F*	*df*	*p*‐Value
Oviposition probability	*M*. *celadussa*	Host plant identity	3.35	1	.067
		Elevation	0.48	1	.490
		Host plant identity × Elevation	1.94	1	.163
		Residuals		28	
	*Z*. *filipendulae*	Host plant identity	6.02	1	**.014**
		Elevation	0.30	1	.581
		Host plant identity × Elevation	1.19	1	.275
		Residuals		16	
Number of egg clutches	*M*. *celadussa*	Host plant identity	4.94	1	**.026**
		Elevation	1.94	1	.164
		Host plant identity × Elevation	0.16	1	.690
		Residuals		28	
	*Z*. *filipendulae*	Host plant identity	7.71	1	**.005**
		Elevation	1.83	1	.176
		Host plant identity × Elevation	0.48	1	.489
		Residuals		16	
Caterpillar preference	*M*. *celadussa*	Host plant identity	4.62	1	**.032**
		*n*		39	
	*Z*. *filipendulae*	Host plant identity	5.40	1	**.020**
		*n*		40	
Caterpillar growth rate	*M*. *celadussa*	Host plant identity	0.02	1	.893
		Elevation	6.70	1	**.011**
		Host plant identity × Elevation	2.61	1	.109
		Residuals		126	
	*Z*. *filipendulae*	Host plant identity	0.00	1	.985
		Elevation	2.71	1	.104
		Host plant identity × Elevation	6.24	1	**.014**
		Residuals		73	
Pupation rate	*M*. *celadussa*	Host plant identity	9.18	1	**.002**
		Residuals		55	
Wing size	*M*. *celadussa*	Host plant identity	7.33	1	**.012**
		Residuals		26	

*Note*: Effects of host plant identity and elevation on oviposition probability, number of egg clutches, caterpillar preference and pupation rate were tested by performing Chi^2^ test, while effects on caterpillar growth and wing size were tested using *F*‐tests. Significant effects are indicated in bold (*p*‐value).

### 3.2 Caterpillar preference

When caterpillars could choose, they consumed larger leaf areas of low‐ compared to high‐elevation host plants (Table 1). Caterpillars of *M*. *celadussa* consumed 21% more leaf area on *P*. *lanceolata* compared to *P*. *atrata* (Figure 2e; Table 1), while leaf consumption was 29% higher on *L*. *corniculatus* compared to *L*. *alpinus* for *Z*. *filipendulae* caterpillars (Figure 2f; Table 1).

### 3.3 Caterpillar performance

Host plant identity had no impact on caterpillar growth rate (Figure 2g,h; Table 1). In contrast, the elevation where food plants were translocated impacted the caterpillar growth rate for *M*. *celadussa*, where the growth rate was 34% lower for caterpillars feeding on *P*. *atrata* growing under warmer temperatures at low elevation compared to caterpillars feeding on *P*. *atrata* growing under cooler temperatures at high elevation. Inversely, for *Z*. *filipendulae*, the growth rate was 1.9× higher for caterpillars feeding on *L*. *alpinus* growing at low elevation compared to caterpillars feeding on *L*. *alpinus* growing at high elevation (Figure 2g, Table 1).

### 3.4 Pupation rate and wing size for *M*. *celadussa*

The pupation rate was 4.4× higher for *M*. *celadussa* caterpillars feeding on *P*. *lanceolata* compared to those feeding on *P*. *atrata* (Figure 2i; Table 1). Moreover, host plant identity also impacted wing size for *M*. *celadussa*, which was 13% smaller for individuals reared on *P*. *atrata* compared to those reared on *P*. *lanceolata* (Figure 2j; Table 1).

### 3.5 Secondary metabolites of plants

**FIGURE 3:**
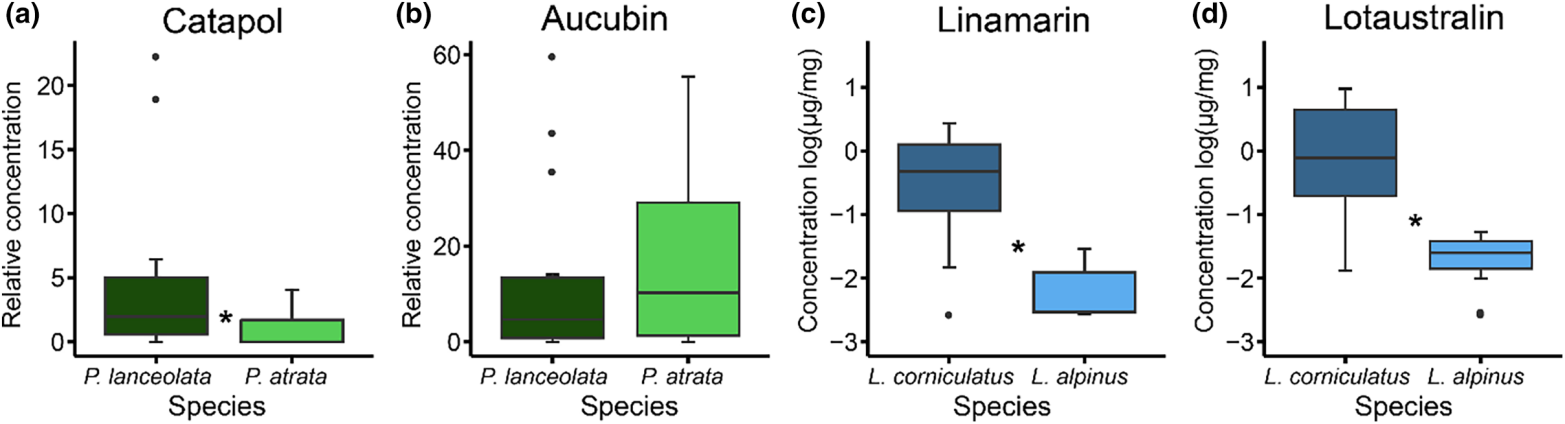
Relative concentrations of catapol and aucubin (a and b) in *Plantago lanceolata* and *Plantago atrata* and absolute concentrations of linamarin and lotaustralin (c and d) in *Lotus corniculatus* and *Lotus alpinus*. Bold, horizontal lines show medians, error bars illustrate distributions of the first to fourth quantiles, and points represent outliers. Asterisks indicate significant differences (*p* < .05) which were calculated using *F*‐tests. Note that effects of elevation are not illustrated here, as translocation at experimental sites (1500/2150 m) had no impact on chemical defence compounds. For effects of elevation on chemical defence compounds, see Figure S8 and Table S7.

Relative concentrations of catapol were 4.6× higher in *P*. *lanceolata* compared to *P*. *atrata*, while no differences in relative aucubin concentrations were detected (Figure 3a,b, Table S7). Absolute concentrations of linamarin and lotaustralin were 144× and 98× (Figure 3c,d, Table S7) higher in *L*. *corniculatus* compared to *L*. *alpinus*. Elevation where plants were translocated had no impact on any of the compounds analysed here (Table S7).

## 4 DISCUSSION

### 4.1 Preference towards ovipositing and feeding on current low‐elevation host plants

Assessing whether specialised low‐elevation insect herbivores can establish novel host plants at higher elevations is crucial to understanding their chances to persist under future scenarios of climate change. Here, we revealed that *M*. *celadussa* and *Z*. *filipendulae* can oviposit and feed on the congeneric alpine plant species, suggesting that shifts from current low‐ to novel high‐elevation host plants under climate change are possible. However, as hypothesised, we found that both species prefer to oviposit and lay more egg clutches, and their caterpillars prefer to feed on current low‐ rather than novel high‐elevation host plants. Chemical cues play important roles in dictating the discrimination between current and potentially novel host plants (Gamberale‐Stille et al., 2014; Ikeura et al., 2010; Ozaki et al., 2011). Accordingly, previous studies have shown that host plant preference for *Z*. *filipendulae* (Zagrobelny et al., 2007, 2014), as well as congeneric species closely related to *M*. *celadussa* (Nieminen et al., 2003; Reudler Talsma et al., 2008), is driven by the presence and concentration of toxic secondary metabolites. For example, feeding on current low‐elevation host plants may be preferred as caterpillars hatching and feeding on more chemically potent plants can sequester higher concentrations of secondary metabolites, toxic for predators and parasitoids (Biere et al., 2004; Bradley et al., 2018; Nieminen et al., 2003). Consequently, preference towards ovipositing and feeding on low‐elevation host plants was not inhibited by higher levels of chemical defences. Instead, possessing higher concentrations of chemical defence compounds could be associated with strengthened resistance against natural enemies via sequestration. Hence, higher concentrations of these molecules could indeed function as oviposition and feeding stimuli for the Lepidoptera species studied here (Bowers, 1983; Pereyra & Bowers, 1988). Future work should be devoted to elucidating the consequences of shifts in host plant identity on the ability of insect herbivores to cope with natural enemies across different elevations (Bruno et al., 2023).

While previous studies show that oviposition probability and success of Lepidoptera species increase under warmer temperatures (Berger et al., 2008; Saastamoinen & Hanski, 2008), in our system, climatic conditions had no impact on oviposition. Specifically, whether *M*. *celadussa* and *Z*. *filipendulae* interacted with low‐ and high‐elevation host plants under warmer climates at low elevation or cooler climates at high elevation neither impacted the preference nor the oviposition success. These results indicate that indirect effects of climate change, reflected in shifts in host plant identity following asynchronous upslope migration of specialised insects and their low‐elevation host plants, may play a larger role in shaping Lepidoptera females' behavioural responses than direct effects of climate. Hence, this study is in line with many others (Alexander et al., 2015; Descombes, Pitteloud, et al., 2020; Nomoto & Alexander, 2021), highlighting the importance of considering the effects of altered species interactions to reliably predict consequences for natural populations facing climate change.

### 4.2 Host plant identity has no impact on caterpillar performance

Contrary to general expectations that feeding on novel host species affects the performance of insects (Ali & Agrawal, 2012; Pelini et al., 2009), we found that caterpillar growth was independent of host plant identity. A potential explanation for this finding is that differences in plant quality were insufficient to be detected within the timeframe of our experiment, as we only monitored caterpillar growth during two instars. Possibly, growth occurring during earlier or later instars may better capture differences in caterpillar growth rates, while monitoring growth during only two developmental stages as in this study may be insufficient to detect the effects of host plant identity on caterpillar growth rates.

Growth rate for caterpillars was lower for *M*. *celadussa* while higher for *Z*. *filipendulae* when feeding on high‐elevation plants translocated to low elevation compared to plants remaining at high elevation. These results could indicate that a rapid increase in temperatures following the translocation of high‐elevation plants to lower elevations may have altered the chemistry of plants and thus changed the palatability of *P*. *atrata* and of *L*. *alpinus* (González‐Teuber et al., 2023). While the production of secondary metabolites in high‐elevation plants remained constant across experimental sites, the translocation across elevations may have altered other phytochemical properties, as well as nutrient contents, of plants, which were not assessed here. For example, Descombes, Kergunteuil, et al. (2020) recorded a shift in the production of 76 untargeted metabolites following the translocation of *P*. *atrata* towards lower elevations. Apart from performing targeted metabolomic analyses, future studies should aim to explore responses using untargeted metabolomics as well as in primary metabolites to better understand how warming per se can change the entire metabolome of host plants and, further, how these potential shifts impact altered plant–insect herbivore interactions.

### 4.3 Novel interactions with high‐elevation host plants reduce pupation rate and wing size for *M*. *celadussa*

While our study suggests that specialised low‐elevation Lepidoptera can colonise and develop on high‐elevation plants, we found that the pupation rate for individuals of *M*. *celadussa* was 4.4× higher if hatching and feeding on its current low‐elevation host plant *P*. *lanceolata* instead of the congeneric high‐elevation plant *P*. *atrata*. In other words, those caterpillars feeding on *P*. *atrata* were more likely to enter diapause at the fourth instar, instead of continuing to grow and entering pupation. Shifts towards entering diapause rather than pupation when exposed to high‐elevation host plants could be explained by a sub‐optimal development due to a less nutritious diet provided by *P*. *atrata* compared to *P*. *lanceolata* (Hunter & McNeil, 1997; Takagi & Miyashita, 2008). Accordingly, lower C:N ratios (i.e. more nitrogen available) in *P*. *lanceolata* than in *P*. *atrata* might have favoured pupation for individuals feeding on the former rather than in the latter species (Loranger et al., 2012). Contrary to rearing performed under controlled conditions, *M*. *celadussa* feeding on *P*. *lanceolata* under natural conditions enters diapause in the fall and only pupates in the spring (LSPN, 1987). When switching to the high‐elevation *P*. *atrata* plants, caterpillars of this species might benefit from a higher diapause rate, which could be more suitable in alpine environments where growing seasons are shorter. Thus, a higher diapause rate may facilitate the ability of *M*. *celadussa* to undergo rapid adaptation in response to changing climates (Buckley & Bridle, 2014).

Previous studies show that Lepidoptera caterpillars experiencing abiotic stress and/or resource limitation develop into adults with smaller wings (Sweeney et al., 1986; Talloen et al., 2009). Apart from abiotic factors, our results indicate shifts in host plant identity also could act to alter wing size, as individuals of *M*. *celadussa* developing on high‐elevation host plants had smaller wings when feeding on *P*. *atrata* instead of *P*. *lanceolata*. As for pupation rate, limited resources due to lower nutrient contents in leaves of high‐elevation plants (Hunter & McNeil, 1997; Takagi & Miyashita, 2008) could explain observed declines in wing size for individuals feeding on *P*. *atrata* instead of *P*. *lanceolata*. As wing size is positively correlated with mobility and flying time in Lepidoptera (Pöyry et al., 2009), *M*. *celadussa* may thus face challenges finding host plants as well as mates due to reduced flight capacity when established at high elevations and forced to oviposit and feed on *P*. *atrata*.

## 5 CONCLUSION

Our study reveals that host plant shifts following upward migration of specialised low‐elevation insect herbivores under climate change are possible, although such shifts could impact their morphology and performance. These changes could alter population dynamics and affect the ability of populations to respond to rapid climate change. Despite the challenges, our findings emphasise the potential for these species to adapt over multiple generations, highlighting the importance of long‐term evolutionary studies to predict species' responses to climate change.

Yet, to confirm our results, several methodological considerations, caveats, and future perspectives could be brought forward: first, performing realistic simulations of climate change in species' natural environments is challenging, often involving limitations in sample size. For instance, the sample size for the oviposition experiment was smaller for *Z*. *filipendulae* compared to *M*. *celadussa*, as egg clutches were detected in only a third of the experimental cages. A possible explanation for the low oviposition rate of *Z*. *filipendulae* could be that some collected females had already laid their eggs before capture. Second, responses to climate change are likely to be species specific. Here, we explored responses to host plant shifts in only two species, which limits the ability to draw general conclusions about how specialised insects are affected by climate change. Future studies should include a broader range of taxa and larger sample sizes to better understand the consequences for insect herbivores facing novel environments under climate change. Third, in our study, we examined the consequences for *M*. *celadussa* and *Z*. *filipendulae* experiencing a shift from their main host plants at low elevations to their closest high‐elevation relatives. Although *P*. *lanceolata* and *L*. *corniculatus* are the main host plants, neither species is strictly monophagous. As argued in the introduction, it is likely that *M*. *celadussa* and *Z*. *filipendulae* would prefer to establish on species that are closely related, as well as morphologically and chemically similar to their current host plants. However, it is possible that they may encounter other suitable host plant species following upward migration. These potential alternative host plants may increase the chances for these butterflies to successfully establish and persist at high elevations. Performing experiments to explore the ability of *M*. *celadussa* and *Z*. *filipendulae* to establish other potential host plants is important to better predict the impact of climate change on the persistence of these species. Fourth, the complex nature of climate change also involves alterations in various biotic interactions, such as plant–plant competition, which may interact with plant–insect dynamics to shape natural communities. Our controlled experiments simulated extreme climate change scenarios, exposing the two insect species to either current low‐ or novel high‐elevation host plants. Under realistic climate change scenarios, low‐elevation insects are initially expected to encounter both low‐ and high‐elevation host plants when migrating upslope. Our study suggests that low‐elevation insect herbivores are likely to cause more damage to low‐elevation plants by feeding and ovipositing more frequently on current low‐elevation host plants. This could potentially counterbalance the negative impacts of highly competitive low‐elevation plants on high‐elevation species (Alexander et al., 2015), initially mitigating their effects (Kempel et al., 2015). Further research examining the combined impacts of multiple abiotic and biotic factors is crucial to understanding the consequences for species facing climate change.

Overall, these considerations and caveats underscore the need for more comprehensive research to improve our understanding of how insect herbivores will adapt to novel environments under climate change. Future studies should estimate longer‐term evolutionary consequences by conducting experiments across multiple generations to predict species' responses to climate change more accurately.

## AUTHOR CONTRIBUTIONS

**Baptiste Bovay:** Conceptualization (equal); data curation (equal); formal analysis (lead); investigation (equal); methodology (lead); visualization (equal); writing – original draft (equal); writing – review and editing (equal). **Patrice Descombes:** Conceptualization (lead); formal analysis (equal); investigation (equal); methodology (equal); supervision (equal); writing – review and editing (equal). **Yannick Chittaro:** Conceptualization (equal); methodology (equal); writing – review and editing (equal). **Gaétan Glauser:** Formal analysis (equal); writing – review and editing (equal). **Hanna Nomoto:** Conceptualization (equal); data curation (equal); formal analysis (equal); investigation (equal); methodology (equal); supervision (lead); validation (equal); visualization (equal); writing – original draft (equal); writing – review and editing (equal). **Sergio Rasmann:** Conceptualization (lead); formal analysis (equal); funding acquisition (equal); investigation (equal); methodology (equal); project administration (equal); supervision (lead); visualization (equal); writing – original draft (equal); writing – review and editing (equal).

## CONFLICT OF INTEREST STATEMENT

The authors declare no conflict of interest.

## STATEMENT OF INCLUSION

Our study brings together authors from different disciplines, including scientists specialised in conservation biology, chemical ecology and functional ecology. The authors were engaged early on with the research and study design to ensure that the diverse sets of perspectives they represent were considered from the onset. Importantly, this study was conducted in close contact with farmers and local people in the region in which the study was carried out, and special efforts were made to discuss and present the study to local stakeholders.

## Supporting information


Appendix S1.

## Data Availability

The data supporting the results (with the exception of occurrence data which are protected) and the code are archived on GitHub (https://github.com/LEF‐UNINE/butterflies_climate_change).

## References

[ece311596-bib-0001] Alexander, J. M. , Chalmandrier, L. , Lenoir, J. , Burgess, T. I. , Essl, F. , Haider, S. , Kueffer, C. , McDougall, K. , Milbau, A. , & Nuñez, M. A. (2018). Lags in the response of mountain plant communities to climate change. Global Change Biology, 24(2), 563–579. 10.1111/gcb.13976 29112781 PMC5813787

[ece311596-bib-0002] Alexander, J. M. , Diez, J. M. , & Levine, J. M. (2015). Novel competitors shape species' responses to climate change. Nature, 525(7570), 515–518. 10.1038/nature14952 26374998

[ece311596-bib-0003] Ali, J. G. , & Agrawal, A. A. (2012). Specialist versus generalist insect herbivores and plant defense. Trends in Plant Science, 17(5), 293–302. 10.1016/j.tplants.2012.02.006 22425020

[ece311596-bib-0004] Andrew, N. R. , & Hughes, L. (2004). Species diversity and structure of phytophagous beetle assemblages along a latitudinal gradient: Predicting the potential impacts of climate change. Ecological Entomology, 29(5), 527–542. 10.1111/j.0307-6946.2004.00639.x

[ece311596-bib-0005] Ash, J. D. , Givnish, T. J. , & Waller, D. M. (2017). Tracking lags in historical plant species' shifts in relation to regional climate change. Global Change Biology, 23(3), 1305–1315. 10.1111/gcb.13429 27416325

[ece311596-bib-0006] Becerra, J. X. , Noge, K. , & Venable, D. L. (2009). Macroevolutionary chemical escalation in an ancient plant–herbivore arms race. Proceedings of the National Academy of Sciences, 106(43), 18062–18066. 10.1073/pnas.0904456106 PMC2775328 19706441

[ece311596-bib-0007] Berg, M. P. , Toby Kiers, E. , Driessen, G. , van der Heijden, M. , Kooi, B. W. , Kuenen, F. , Liefting, M. , Verhoef, H. A. , & Ellers, J. (2010). Adapt or disperse: Understanding species persistence in a changing world. Global Change Biology, 16(2), 587–598. 10.1111/j.1365-2486.2009.02014.x

[ece311596-bib-0008] Berger, D. , Walters, R. , & Gotthard, K. (2008). What limits insect fecundity? Body size‐and temperature‐dependent egg maturation and oviposition in a butterfly. Functional Ecology, 22(3), 523–529. 10.1111/j.1365-2435.2008.01392.x

[ece311596-bib-0009] Biere, A. , Marak, H. B. , & Van Damme, J. M. M. (2004). Plant chemical defense against herbivores and pathogens: Generalized defense or trade‐offs? Oecologia, 140(3), 430–441. 10.1007/s00442-004-1603-6 15146326

[ece311596-bib-0010] Bowers, M. D. (1983). The role of iridoid glycosides in host‐plant specificity of checkerspot butterflies. Journal of Chemical Ecology, 9, 475–493. 10.1007/BF00990220 24407455

[ece311596-bib-0011] Bradley, L. E. , Kelly, C. A. , & Bowers, M. D. (2018). Host plant suitability in a specialist herbivore, *Euphydryas anicia* (Nymphalidae): Preference, performance and sequestration. Journal of Chemical Ecology, 44(11), 1051–1057. 10.1007/s10886-018-1012-7 30175378

[ece311596-bib-0012] Braschler, B. , & Hill, J. K. (2007). Role of larval host plants in the climate‐driven range expansion of the butterfly Polygonia c‐album. Journal of Animal Ecology, 76(3), 415–423. 10.1111/J.1365-2656.2007.01217.X 17439459

[ece311596-bib-0013] Bruno, P. , Arce, C. C. M. , Machado, R. A. R. , Besomi, G. , Spescha, A. , Glauser, G. , Jaccard, C. , Benrey, B. , & Turlings, T. C. J. (2023). Sequestration of cucurbitacins from cucumber plants by *Diabrotica balteata* larvae provides little protection against biological control agents. Journal of Pest Science, 96(3), 1061–1075. 10.1007/s10340-022-01568-3 37181825 PMC10169900

[ece311596-bib-0014] Buckley, J. , & Bridle, J. R. (2014). Loss of adaptive variation during evolutionary responses to climate change. Ecology Letters, 17(10), 1316–1325. 10.1111/ele.12340 25104062

[ece311596-bib-0015] Chen, I.‐C. , Shiu, H.‐J. , Benedick, S. , Holloway, J. D. , Chey, V. K. , Barlow, H. S. , Hill, J. K. , & Thomas, C. D. (2009). Elevation increases in moth assemblages over 42 years on a tropical mountain. Proceedings of the National Academy of Sciences, 106(5), 1479–1483. 10.1073/pnas.0809320106 PMC2635813 19164573

[ece311596-bib-0016] Clarke, H. E. (2022). A provisional checklist of European butterfly larval foodplants. Nota Lepidopterologica, 45(2001), 139–167. 10.3897/NL.45.72017

[ece311596-bib-0017] Corlett, R. T. , & Westcott, D. A. (2013). Will plant movements keep up with climate change? Trends in Ecology & Evolution, 28(8), 482–488. 10.1016/j.tree.2013.04.003 23721732

[ece311596-bib-0018] Dahlke, F. T. , Wohlrab, S. , Butzin, M. , & Pörtner, H.‐O. (2020). Thermal bottlenecks in the life cycle define climate vulnerability of fish. Science, 369(6499), 65–70. 10.1126/science.aaz3658 32631888

[ece311596-bib-0019] Descombes, P. , Kergunteuil, A. , Glauser, G. , Rasmann, S. , & Pellissier, L. (2020). Plant physical and chemical traits associated with herbivory in situ and under a warming treatment. Journal of Ecology, 108(2), 733–749. 10.1111/1365-2745.13286

[ece311596-bib-0020] Descombes, P. , Marchon, J. , Pradervand, J. N. , Bilat, J. , Guisan, A. , Rasmann, S. , & Pellissier, L. (2017). Community‐level plant palatability increases with elevation as insect herbivore abundance declines. Journal of Ecology, 105(1), 142–151. 10.1111/1365-2745.12664

[ece311596-bib-0021] Descombes, P. , Pitteloud, C. , Glauser, G. , Defossez, E. , Kergunteuil, A. , Allard, P. M. , Rasmann, S. , & Pellissier, L. (2020). Novel trophic interactions under climate change promote alpine plant coexistence. Science, 370(6523), 1469–1473. 10.1126/science.abd7015 33335062

[ece311596-bib-0022] Doak, D. F. , & Morris, W. F. (2010). Demographic compensation and tipping points in climate‐induced range shifts. Nature, 467(7318), 959–962. 10.1038/nature09439 20962844

[ece311596-bib-0023] Ehrlich, P. R. , & Raven, P. H. (1964). Butterflies and plants: A study in coevolution. Evolution, 18(4), 586–608. 10.2307/2406212

[ece311596-bib-0024] Fahey, J. W. , Zalcmann, A. T. , & Talalay, P. (2001). The chemical diversity and distribution of glucosinolates and isothiocyanates among plants. Phytochemistry, 56(1), 5–51. 10.1016/S0031-9422(00)00316-2 11198818

[ece311596-bib-0025] Farrell, B. , & Mitter, C. (1998). The timing of insect/plant diversification: Might Tetraopes (Coleoptera: Cerambycidae) and Asclepias (Asclepiadaceae) have co‐evolved? Biological Journal of the Linnean Society, 63(4), 553–577. 10.1111/j.1095-8312.1998.tb00329.x

[ece311596-bib-0026] Forister, M. L. , McCall, A. C. , Sanders, N. J. , Fordyce, J. A. , Thorne, J. H. , O'Brien, J. , Waetjen, D. P. , & Shapiro, A. M. (2010). Compounded effects of climate change and habitat alteration shift patterns of butterfly diversity. Proceedings of the National Academy of Sciences, 107(5), 2088–2092. 10.1073/pnas.0909686107 PMC2836664 20133854

[ece311596-bib-0027] Fox, J. , & Weisberg, S. (2019). An {R} companion to applied regression (3rd ed.). Sage. https://socialsciences.mcmaster.ca/jfox/Books/Companion/

[ece311596-bib-0028] Futuyma, D. J. , & Moreno, G. (1988). The evolution of ecological specialization. Annual Review of Ecology and Systematics, 19(1), 207–233. 10.1146/annurev.es.19.110188.001231

[ece311596-bib-0029] Gamberale‐Stille, G. , Söderlind, L. , Janz, N. , & Nylin, S. (2014). Host plant choice in the comma butterfly‐larval choosiness may ameliorate effects of indiscriminate oviposition. Insect Science, 21(4), 499–506. 10.1111/1744-7917.12059 24006353

[ece311596-bib-0030] Gassmann, A. , Levy, A. , Tran, T. , & Futuyma, D. (2006). Adaptations of an insect to a novel host plant: A phylogenetic approach. Functional Ecology, 20(3), 478–485. 10.1111/j.1365-2435.2006.01118.x

[ece311596-bib-0032] Gilman, S. E. , Urban, M. C. , Tewksbury, J. , Gilchrist, G. W. , & Holt, R. D. (2010). A framework for community interactions under climate change. Trends in Ecology & Evolution, 25(6), 325–331. 10.1016/j.tree.2010.03.002 20392517

[ece311596-bib-0033] Glassmire, A. E. , Jeffrey, C. S. , Forister, M. L. , Parchman, T. L. , Nice, C. C. , Jahner, J. P. , Wilson, J. S. , Walla, T. R. , Richards, L. A. , & Smilanich, A. M. (2016). Intraspecific phytochemical variation shapes community and population structure for specialist caterpillars. New Phytologist, 212(1), 208–219. 10.1111/nph.14038 27279551 PMC5089596

[ece311596-bib-0034] González‐Teuber, M. , Palma‐Onetto, V. , Aguirre, C. , Ibáñez, A. J. , & Mithöfer, A. (2023). Climate change‐related warming‐induced shifts in leaf chemical traits favor nutrition of the specialist herbivore *Battus polydamas* archidamas. Frontiers in Ecology and Evolution, 11, 1152489. 10.3389/fevo.2023.1152489

[ece311596-bib-0035] Griffin, W. J. , & Lin, G. D. (2000). Chemotaxonomy and geographical distribution of tropane alkaloids. Phytochemistry, 53(6), 623–637. 10.1016/S0031-9422(99)00475-6 10746874

[ece311596-bib-0036] Guenin, R. (2023). Bildatlas der Rot‐ und Grünwidderchen des Alpenraums (Zygaenidae: Zygaeninae, Procridinae, Chalcosiinae). Contribution to Natural History, 39, 1–1007.

[ece311596-bib-0037] Hanspach, J. , Schweiger, O. , Kühn, I. , Plattner, M. , Pearman, P. B. , Zimmermann, N. E. , & Settele, J. (2014). Host plant availability potentially limits butterfly distributions under cold environmental conditions. Ecography, 37(3), 301–308. 10.1111/j.1600-0587.2013.00195.x

[ece311596-bib-0038] Harrison, J. G. , Gompert, Z. , Fordyce, J. A. , Buerkle, C. A. , Grinstead, R. , Jahner, J. P. , Mikel, S. , Nice, C. C. , Santamaria, A. , & Forister, M. L. (2016). The many dimensions of diet breadth: Phytochemical, genetic, behavioral, and physiological perspectives on the interaction between a native herbivore and an exotic host. PLoS One, 11(2), e0147971. 10.1371/journal.pone.0147971 26836490 PMC4737494

[ece311596-bib-0039] Hartig, F. (2022). DHARMa: Residual Diagnostics for Hierarchical (Multi‐Level/Mixed) Regression Models. https://CRAN.R‐project.org/package=DHARMa

[ece311596-bib-0040] HilleRisLambers, J. , Harsch, M. A. , Ettinger, A. K. , Ford, K. R. , & Theobald, E. J. (2013). How will biotic interactions influence climate change–induced range shifts? Annals of the New York Academy of Sciences, 1297(1), 112–125. 10.1111/nyas.12182 23876073

[ece311596-bib-0041] Honda, K. (1995). Chemical basis of differential oviposition by lepidopterous insects. Archives of Insect Biochemistry and Physiology, 30(1), 1–23. 10.1002/arch.940300102

[ece311596-bib-0042] Hunter, M. D. , & McNeil, J. N. (1997). Host‐plant quality influences diapause and voltinism in a polyphagous insect herbivore. Ecology, 78(4), 977–986. 10.1890/0012-9658(1997)078[0977:HPQIDA]2.0.CO;2

[ece311596-bib-0043] Ikeura, H. , Kobayashi, F. , & Hayata, Y. (2010). How do *Pieris rapae* search for Brassicaceae host plants? Biochemical Systematics and Ecology, 38(6), 1199–1203. 10.1016/j.bse.2010.12.007

[ece311596-bib-0044] IPBES . (2019). Summary for policymakers of the global assessment report on biodiversity and ecosystem services of the Intergovernmental Science‐Policy Platform on Biodiversity and Ecosystem Services. S. Díaz, J. Settele, E. S. Brondízio, H. T. Ngo, M. Guèze, J. Agard, A. Arneth, P. Balvanera, K. A. Brauman, S. H. M. Butchart, K. M. A. Chan, L. A. Garibaldi, K. Ichii, J. Liu, S. M. Subramanian, G. F. Midgley, P. Miloslavich, Z. Molnár, D. Obura, A. Pfaff, S. Polasky, A. Purvis, J. Razzaque, B. Reyers, R. Roy Chowdhury, Y. J. Shin, I. J. Visseren‐Hamakers, K. J. Willis, & C. N. Zayas (Eds.). 10.5281/zenodo.3553579

[ece311596-bib-0045] IPCC . (2014). Climate change 2014: Synthesis report. Contribution of working groups I, II and III to the fifth assessment report of the intergovernmental panel on climate change. IPCC.

[ece311596-bib-0046] IPCC . (2023). Climate change 2023: Synthesis report. Contribution of working groups I, II and III to the sixth assessment report of the intergovernmental panel on climate change. IPCC.

[ece311596-bib-0047] Jeschke, V. , Kearney, E. E. , Schramm, K. , Kunert, G. , Shekhov, A. , Gershenzon, J. , & Vassão, D. G. (2017). How glucosinolates affect generalist lepidopteran larvae: Growth, development and glucosinolate metabolism. Frontiers in Plant Science, 8, 1995. 10.3389/fpls.2017.01995 29209354 PMC5702293

[ece311596-bib-0048] Kempel, A. , Razanajatovo, M. , Stein, C. , Unsicker, S. B. , Auge, H. , Weisser, W. W. , Fischer, M. , & Prati, D. (2015). Herbivore preference drives plant community composition. Ecology, 96(11), 2923–2934. 10.1890/14-2125.1 27070012

[ece311596-bib-0049] Kerner, J. M. , Krauss, J. , Maihoff, F. , Bofinger, L. , & Classen, A. (2023). Alpine butterflies want to fly high: Species and communities shift upwards faster than their host plants. Ecology, 104(1), e3848. 10.1002/ecy.3848 36366785

[ece311596-bib-0050] Knolhoff, L. M. , & Heckel, D. G. (2014). Behavioral assays for studies of host plant choice and adaptation in herbivorous insects. Annual Review of Entomology, 59, 263–278. 10.1146/annurev-ento-011613-161945 24160429

[ece311596-bib-0051] Kuznetsova, A. , Brockhoff, P. B. , & Christensen, R. H. B. (2017). {lmerTest} Package: Tests in linear mixed effects models. Journal of Statistical Software, 82(13), 1–26. 10.18637/jss.v082.i13

[ece311596-bib-0052] Lauber, K. , Wagner, G. , & Gygax, A. (2018). *Flora Helvetica* (Haupt ed.).

[ece311596-bib-0053] Lenth, R. V. (2022). emmeans: Estimated Marginal Means, aka Least‐Squares Means. https://CRAN.R‐project.org/package=emmeans

[ece311596-bib-0054] Loranger, J. , Meyer, S. T. , Shipley, B. , Kattge, J. , Loranger, H. , Roscher, C. , & Weisser, W. W. (2012). Predicting invertebrate herbivory from plant traits: Evidence from 51 grassland species in experimental monocultures. Ecology, 93(12), 2674–2682. 10.1890/12-0328.1 23431597

[ece311596-bib-0055] LSPN . (1987). *Les papillons de jour et leurs biotopes*. *Espèces*. *Dangers qui les menacent*. *Protection* (LSPN, Ed.).

[ece311596-bib-0056] LSPN . (1999). *Les papillons et leurs biotopes*. *Espèces – Dangers qui les menacent – Protection*. *Suisse et régions limitrophes*. *Tome 2* (LSPN, Ed.).

[ece311596-bib-0057] Merrill, R. M. , Gutiérrez, D. , Lewis, O. T. , Gutiérrez, J. , Díez, S. B. , & Wilson, R. J. (2008). Combined effects of climate and biotic interactions on the elevational range of a phytophagous insect. Journal of Animal Ecology, 145‐155, 145–155. 10.1111/j.1365-2656.2007.01303.X 18177334

[ece311596-bib-0058] Moir, M. L. , Hughes, L. , Vesk, P. A. , & Leng, M. C. (2014). Which host‐dependent insects are most prone to coextinction under changed climates? Ecology and Evolution, 4(8), 1295–1312. 10.1002/ece3.1021 24834327 PMC4020690

[ece311596-bib-0059] Näsvall, K. , Wiklund, C. , Mrazek, V. , Künstner, A. , Talla, V. , Busch, H. , Vila, R. , & Backström, N. (2021). Host plant diet affects growth and induces altered gene expression and microbiome composition in the wood white (*Leptidea sinapis*) butterfly. Molecular Ecology, 30(2), 499–516. 10.1111/mec.15745 33219534 PMC7839524

[ece311596-bib-0060] Nieminen, M. , Suomi, J. , Van Nouhuys, S. , Sauri, P. , & Riekkola, M. L. (2003). Effect of iridoid glycoside content on oviposition host plant choice and parasitism in a specialist herbivore. Journal of Chemical Ecology, 29(4), 823–844. 10.1023/A:1022923514534 12775146

[ece311596-bib-0061] Nishida, R. (2014). Chemical ecology of insect‐plant interactions: Ecological significance of plant secondary metabolites. Bioscience, Biotechnology, and Biochemistry, 78(1), 1–13. 10.1080/09168451.2014.877836 25036477

[ece311596-bib-0062] Nomoto, H. A. , & Alexander, J. M. (2021). Drivers of local extinction risk in alpine plants under warming climate. Ecology Letters, 24(6), 1157–1166. 10.1111/ele.13727 33780124 PMC7612402

[ece311596-bib-0063] Nooten, S. S. , & Hughes, L. (2017). The power of the transplant: Direct assessment of climate change impacts. Climatic Change, 144, 237–255. 10.1007/s10584-017-2037-6

[ece311596-bib-0064] Ozaki, K. , Ryuda, M. , Yamada, A. , Utoguchi, A. , Ishimoto, H. , Calas, D. , Marion‐Poll, F. , Tanimura, T. , & Yoshikawa, H. (2011). A gustatory receptor involved in host plant recognition for oviposition of a swallowtail butterfly. Nature Communications, 2(1), 542. 10.1038/ncomms1548 22086342

[ece311596-bib-0065] Paolucci, P. (2013). Butterflies and burnets of the Alps and their larvae, pupae and cocoons. WBA handbooks (Vol. 42), WBA PROJECT SRL impresa sociale. 10.1080/00094056.1965.10729084

[ece311596-bib-0066] Parmesan, C. , & Yohe, G. (2003). A globally coherent fingerprint of climate change impacts across natural systems. Nature, 421(6918), 37–42. 10.1038/nature01286 12511946

[ece311596-bib-0067] Parolo, G. , & Rossi, G. (2008). Upward migration of vascular plants following a climate warming trend in the Alps. Basic and Applied Ecology, 9(2), 100–107. 10.1016/j.baae.2007.01.005

[ece311596-bib-0068] Parry, D. , & Goyer, R. A. (2004). Variation in the suitability of host tree species for geographically discrete populations of forest tent caterpillar. Environmental Entomology, 33(5), 1477–1487. 10.1603/0046-225X-33.5.1477

[ece311596-bib-0069] Pauli, H. , Gottfried, M. , Dullinger, S. , Abdaladze, O. , Akhalkatsi, M. , Alonso, J. L. B. , Coldea, G. , Dick, J. , Erschbamer, B. , Calzado, R. F. , Ghosn, D. , Holten, J. I. , Kanka, R. , Kazakis, G. , Kollár, J. , Larsson, P. , Moiseev, P. , Moiseev, D. , Molau, U. , … Grabherr, G. (2012). Recent plant diversity changes on Europe's mountain summits. Science, 336(6079), 353–355. 10.1126/science.1219033 22517860

[ece311596-bib-0070] Pelini, S. L. , Dzurisin, J. D. , Prior, K. M. , Williams, C. M. , Marsico, T. D. , Sinclair, B. J. , & Hellmann, J. J. (2009). Translocation experiments with butterflies reveal limits to enhancement of poleward populations under climate change. Proceedings of the National Academy of Sciences of the United States of America, 106(27), 11160–11165. 10.1073/pnas.0900284106 19549861 PMC2708697

[ece311596-bib-0071] Pelini, S. L. , Keppel, J. A. , Kelley, A. E. , & Hellmann, J. J. (2010). Adaptation to host plants may prevent rapid insect responses to climate change. Global Change Biology, 16(11), 2923–2929. 10.1111/j.1365-2486.2010.02177.x

[ece311596-bib-0072] Pereyra, P. C. , & Bowers, M. D. (1988). Iridoid glycosides as oviposition stimulants for the buckeye butterfly, Junonia coenia (Nymphalidae). Journal of Chemical Ecology, 14, 917–928. 10.1007/BF01018783 24276141

[ece311596-bib-0073] Pöyry, J. , Luoto, M. , Heikkinen, R. K. , Kuussaari, M. , & Saarinen, K. (2009). Species traits explain recent range shifts of Finnish butterflies. Global Change Biology, 15(3), 732–743. 10.1111/j.1365-2486.2008.01789.x

[ece311596-bib-0074] R Core Team . (2021). R: A Language and Environment for Statistical Computing. https://www.r‐project.org/

[ece311596-bib-0075] Radchuk, V. , Turlure, C. , & Schtickzelle, N. (2013). Each life stage matters: The importance of assessing the response to climate change over the complete life cycle in butterflies. Journal of Animal Ecology, 82(1), 275–285. 10.1111/j.1365-2656.2012.02029.x 22924795

[ece311596-bib-0076] Reudler Talsma, J. H. , Torri, K. , & van Nouhuys, S. (2008). Host plant use by the heath fritillary butterfly, *Melitaea athalia*: Plant habitat, species and chemistry. Arthropod‐Plant Interactions, 2(2), 63–75. 10.1007/s11829-008-9039-2

[ece311596-bib-0077] Richman, S. K. , Levine, J. M. , Stefan, L. , & Johnson, C. A. (2020). Asynchronous range shifts drive alpine plant–pollinator interactions and reduce plant fitness. Global Change Biology, 26(5), 3052–3064. 10.1007/s11412-020-09326-2 32061109

[ece311596-bib-0078] Rödder, D. , Schmitt, T. , Gros, P. , Ulrich, W. , & Habel, J. C. (2021). Climate change drives mountain butterflies towards the summits. Scientific Reports, 11(1), 1–12. 10.1038/s41598-021-93826-0 34257364 PMC8277792

[ece311596-bib-0079] Rønsted, N. , Chase, M. W. , Albach, D. C. , & Bello, M. A. (2002). Phylogenetic relationships within Plantago (Plantaginaceae): Evidence from nuclear ribosomal ITS and plastid trnL‐F sequence data. Botanical Journal of the Linnean Society, 139(4), 323–338. 10.1046/j.1095-8339.2002.00070.x

[ece311596-bib-0080] Rønsted, N. , Göbel, E. , Franzyk, H. , Jensen, S. R. , & Olsen, C. E. (2000). Chemotaxonomy of Plantago. Iridoid glucosides and caffeoyl phenylethanoid glycosides. Phytochemistry, 55(4), 337–348. 10.1016/S0031-9422(00)00306-X 11117882

[ece311596-bib-0081] RStudio Team . (2021). RStudio: Integrated Development Environment for R. http://www.rstudio.com/

[ece311596-bib-0082] Saastamoinen, M. , & Hanski, I. (2008). Genotypic and environmental effects on flight activity and oviposition in the Glanville fritillary butterfly. The American Naturalist, 171(6), 701–712. 10.1086/587531 18419339

[ece311596-bib-0083] Schneider, C. A. , Rasband, W. S. , & Eliceiri, K. W. (2012). NIH image to ImageJ: 25 years of image analysis. Nature Methods, 9(7), 671–675. 10.1038/nmeth.2089 22930834 PMC5554542

[ece311596-bib-0084] Sun, Z. , Zhang, K. , Chen, C. , Wu, Y. , Tang, Y. , Georgiev, M. I. , Zhang, X. , Lin, M. , & Zhou, M. (2018). Biosynthesis and regulation of cyanogenic glycoside production in forage plants. Applied Microbiology and Biotechnology, 102(1), 9–16. 10.1007/s00253-017-8559-z 29022076

[ece311596-bib-0085] Sweeney, B. W. , Vannote, R. L. , & Dodds, P. J. (1986). Effects of temperature and food quality on growth and development of a mayfly, *Leptophlebia intermedia* . Canadian Journal of Fisheries and Aquatic Sciences, 43(1), 12–18. 10.1139/f86-002

[ece311596-bib-0086] Takagi, S. , & Miyashita, T. (2008). Host plant quality influences diapause induction of *Byasa alcinous* (lepidoptera: Papilionidae). Annals of the Entomological Society of America, 101(2), 392–396. 10.1603/0013-8746(2008)101[392:HPQIDI]2.0.CO;2

[ece311596-bib-0087] Talloen, W. , Van Dongen, S. , Van Dyck, H. , & Lens, L. (2009). Environmental stress and quantitative genetic variation in butterfly wing characteristics. Evolutionary Ecology, 23(3), 473–485. 10.1007/s10682-008-9246-4

[ece311596-bib-0088] Tito, R. , Vasconcelos, H. L. , & Feeley, K. J. (2020). Mountain ecosystems as natural laboratories for climate change experiments. Frontiers in Forests and Global Change, 3(38), 1–8. 10.3389/ffgc.2020.00038

[ece311596-bib-0089] Urban, M. C. , Tewksbury, J. J. , & Sheldon, K. S. (2012). On a collision course: Competition and dispersal differences create no‐analogue communities and cause extinctions during climate change. Proceedings of the Royal Society B: Biological Sciences, 279(1735), 2072–2080. 10.1098/rspb.2011.2367 PMC3311897 22217718

[ece311596-bib-0090] Vitasse, Y. , Ursenbacher, S. , Klein, G. , Bohnenstengel, T. , Chittaro, Y. , Delestrade, A. , Monnerat, C. , Rebetez, M. , Rixen, C. , Strebel, N. , Schmidt, B. R. , Wipf, S. , Wohlgemuth, T. , Yoccoz, N. G. , & Lenoir, J. (2021). Phenological and elevational shifts of plants, animals and fungi under climate change in the European Alps. Biological Reviews, 96(5), 1816–1835. 10.1111/brv.12727 33908168

[ece311596-bib-0091] Walther, G.‐R. , Post, E. , Convey, P. , Menzel, A. , Parmesan, C. , Beebee, T. J. , Fromentin, J.‐M. , Hoegh‐Guldberg, O. , & Bairlein, F. (2002). Ecological responses to recent climate change. Nature, 416(6879), 389–395. 10.1038/416389a 11919621

[ece311596-bib-0092] Warren, M. , Hill, J. , Thomas, J. , Asher, J. , Fox, R. , Huntley, B. , Roy, D. , Telfer, M. , Jeffcoate, S. , & Harding, P. (2001). Rapid responses of British butterflies to opposing forces of climate and habitat change. Nature, 414(6859), 65–69. 10.1038/35102054 11689943

[ece311596-bib-0093] Wermeille, E. , Chittaro, Y. , & Gonseth, Y. (2014). Liste rouge Papillons diurnes et Zygènes. Espèces menacées en Suisse, état 2012. *L'environnement pratique*, *1403*, OFEV and CSCF (Eds.).

[ece311596-bib-0094] Wickham, H. (2016). ggplot2: Elegant graphics for data analysis. Springer‐Verlag New York. https://ggplot2.tidyverse.org

[ece311596-bib-0095] Yamamoto, S. , & Uchida, K. (2018). A generalist herbivore requires a wide array of plant species to maintain its populations. Biological Conservation, 228, 167–174. 10.1016/j.biocon.2018.10.018

[ece311596-bib-0096] Zagrobelny, M. , Bak, S. , Thorn Ekstrøm, C. , Erik Olsen, C. , & Lindberg Møller, B. (2007). The cyanogenic glucoside composition of *Zygaena filipendulae* (Lepidoptera: Zygaenidae) as effected by feeding on wild‐type and transgenic lotus populations with variable cyanogenic glucoside profiles. Insect Biochemistry and Molecular Biology, 37(1), 10–18. 10.1016/j.ibmb.2006.09.008 17175442

[ece311596-bib-0097] Zagrobelny, M. , Olsen, C. E. , Pentzold, S. , Fürstenberg‐Hägg, J. , Jørgensen, K. , Bak, S. , Møller, B. L. , & Motawia, M. S. (2014). Sequestration, tissue distribution and developmental transmission of cyanogenic glucosides in a specialist insect herbivore. Insect Biochemistry and Molecular Biology, 44(1), 44–53. 10.1016/j.ibmb.2013.11.003 24269868

