# Correction to “Adapting to Change: Exploring the Consequences of Climate‐Induced Host Plant Shifts in Two Specialist Lepidoptera Species”

**DOI:** 10.1002/ece3.70357

**Published:** 2024-09-30

Bovay, B., P. Descombes, Y. Chittaro, G. Glauser, H. Nomoto, and S. Rasmann. 2024. “Adapting to Change: Exploring the Consequences of Climate‐Induced Host Plant Shifts in Two Specialist Lepidoptera Species.” *Ecology and Evolution* 14, no. 6: e11596.

In the “Data availability statement,” the link to the data repository was incorrect. The correct link is https://doi.org/10.17605/OSF.IO/HRYQS.

We apologize for this error.

