# Supplementary material for: Adapting to change: Exploring the consequences of climate‐induced host plant shifts in two specialist Lepidoptera species
Source: Ecol Evol. 2024 Jun 25;14(6):e11596. doi: 10.1002/ece3.11596 (PMC11199125; doi:10.1002/ece3.11596)
Supplement: Supplementary file 1 — Appendix S1. [file ECE3-14-e11596-s001.docx]

# APPENDIX

# Supplementary Methods

## Sex determination of M. celadussa and Z. filipendulae

Sex determination of *M. celadussa* and *Z. filipendulae* based on the size and shape of individuals’ abdomens was performed immediately after capturing individuals in field. Females have larger, ovoid abdomens, while males have thinner, parallel abdomens.

## Secondary metabolites analyses

A total of 3-9 leaves per individual were collected and placed in a -80 °C freezer (Thermo scientific, Waltham, USA) the week following the field experiment, then freeze-dried for 72 hours using a LyoQuest (Telstar, Azbil Corporation, Tokyo, Japan). 10 (+/- 2) mg of freeze-dried leaf material was milled into powder using an MM400 Retch TissueLyser (Qiagen, Hilden, Germany) for 4 minutes at 30 Hz, and extraction of the secondary metabolites was performed with 1.5 mL 100% methanol for *Plantago* spp., and 1 mL of 70:30% methanol:water for *Lotus* spp. After centrifugation (3 minutes, 14,000 rpm), 200 μl of the supernatant from each sample was transferred to HPLC vials for liquid chromatography-mass spectrometry for *Lotus* spp., while *Plantago* spp. samples were diluted with 800 μl MiliQ water and centrifuged before 200 μl of the supernatant were transferred to HPLC vials.

Secondary metabolites were analysed using ultra-high-performance liquid chromatography-quadrupole time-of-flight mass spectrometry (UHPLC/Q-TOF-MS; Waters, Milford, USA) following methods described in Bakhtiari et al. (2019) and Formenti et al. (2021) for iridoid glycosides (IGs), and Shlichta et al. (2014) for cyanogenic glycosides (CNGs). Absolute concentrations of CNGs (mg/g) were calculated using external calibration prepared from linamarin and lotaustralin standards (Santa Cruz Biotechnology, Santa Cruz, CA, USA) in 70 % methanol at concentrations of 0.2, 1, 5 and 25 µg/mL. As reference standards were absent for IGs, only relative concentrations were obtained for catapol and aucubin. Data was processed using TargetLynx XS (Waters, Milford, USA) where all peaks corresponding to the retention time of the targeted metabolites were individually checked. When peaks were visible, but concentration could not be calculated, we used the value of the detection threshold. Concentrations below the detection threshold were replaced by 0.

*Diapause/pupation determination of M. celadussa*

## For each caterpillar of M. celadussa for which pupation rate was estimated, we recorded whether individuals entered pupation (1) or diapause (0). Caterpillars entering diapause stop growing at 0.2 ± 0.1 g and become brownish. Non-diapausing caterpillars rapidly reach 1.5 g and do not change colour. All diapausing larvae were either released on site of capture of the parents or died.

## Assessments of plant traits

To explore morphological differences between low- and high-elevation host plants, we randomly selected n = 10, 10, 11 and 10 individuals of *P. lanceolata*, *P. atrata*, *L. corniculatus* and *L. alpinus*, respectively, growing at the experimental site situated at low-elevation and n = 11, 8, 9 and 9 individuals of *P. lanceolata*, *P. atrata*, *L. corniculatus* and *L. alpinus*, respectively, growing at the experimental site situated at high-elevation. For all these plants, morphological traits were monitored. Individuals selected were included in the oviposition experiment, except for a few *Plantago* spp. individuals (n = 4, 6 individuals of *P. lanceolata* growing at low- and high-elevation, respectively, and n = 3, 3 individuals of *P. atrata* growing at low- and high-elevation), which, although not exposed to *M. celadussa* and *Z. filipendulae*, had been translocated across elevations. Immediately after the field experiment, we monitored stalk height (stretch height from base to highest structure), number of leaves and number of inflorescences. For *Plantago* spp. we also recorded the number of rosettes, while the number of stems was recorded for *Lotus* spp.

We collected one leaf (the youngest fully expanded leaf) to estimate specific leaf area (SLA). To estimate SLA we scanned leaves and calculated the leaf area with ImageJ (Schneider et al., 2012). Leaves were dried at 40° for 6 days before weighing them using a Stratorius scale (Startorius Lab Instruments GmbH & Co, Göttingen, Germany; 0.1 mg accuracy). SLA was calculated by dividing leaf area (mm^2^) by leaf weight (mg).

Carbon and Nitrogen (C:N) ratios in leaves were estimated on the same leaf samples used to estimate SLA for which amounts of leaf dry matter required to estimate C:N ratio was sufficient (n = 6, 7, 7 and 6 individuals growing at experimental sites at low-elevation for *P. lanceolata*, *P. atrata*, *L. corniculatus* and *L. alpinus*, respectively, and n = 7, 6, 7 and 6 individuals growing at experimental sites at low-elevation for *P. lanceolata*, *P. atrata*, *L. corniculatus* and *L. alpinus*, respectively). To analyse C:N ratios, leaves were milled into powder and 1-3 mg powder was weighed using a Stratorius microscale (Startorius Lab Instruments GmbH & Co, Göttingen, Germany; 0.001 mg accuracy) and placed in a tin capsule. Samples were analysed with an elemental analyser (Flash 2000, CHN-O Analyzer, Thermo Scientific, Waltham, Massachusetts, United States) and C:N ratios were calculated by diving total carbon (C) by the total nitrogen content (N).

## Analysing differences in morphological traits and C:N ratios between host plant identities

Differences in morphological traits between low- and high-elevation host plants were explored by performing Principal Component Analyses (PCA) using the *factoextra* package (Kassambara & Mundt, 2020) including stalk height, number of leaves, number of inflorescences, SLA, number of stems (*Lotus* spp.) and rosettes (*Plantago* spp.) as response variables and host plant identity and elevation as explanatory variable. PCAs were fitted for *Plantago* spp. and *Lotus* spp. separately. Differences in morphological traits between plant species and the elevation were explored by performing permutational multivariate ANOVA (PERMANOVA) analyses with Bray-Curtis distance on PCA models using the *vegan* package (Oksanen *et al.* 2020).

Differences in C:N ratios between host plants were explored by fitting a linear model with C:N ratio as response variable and the interactive effect of host plant identity (*Plantago* spp. and *Lotus* spp. species combined) and elevation as explanatory variables. Significant effects of host plant identity and elevation on C:N ratios were estimated by performing F-tests of fitted models using ANOVA. To test pairwise differences in C:N ratio between host plants, we performed posthoc tests by using the *emmeans* package (Lenth, 2022).

For differences in morphology and C:N ratios between host plant identities, see Figure S6-7 and Table S4-5.

## Modelling of the climatic niches for M. celadussa and Z. filipendulae

Species distributions for *M. celadussa* and *Z. filipendulae* were modelized using all occurrences of each species recorded in Switzerland and stored in the National Data and Information Center on the Swiss Fauna (www.infofauna.ch). Data was filtered so that only observations recorded with a precision of less than 100 m were included. In addition, to reduce special autocorrelation, occurrence data were disaggregated by a distance of 100 m. For *M. celadussa*, we also used some observations of the aggregate *Melitaea athalia* aggr., containing two cryptic species (*M. celadussa* and *M. athalia*). These species occur in two different regions in Switzerland and the cantons of Vaud, Valis, Geneva, Ticino and Fribourg have only populations of *M. celadussa* (Litman et al., 2018), thus we also used the occurrence of the aggregate in these cantons. Species distributions were modelled based on a total of n = 4,858 and 7,293 occurrences for *M. celadussa* and *Z. filipendulae*, respectively.

To model current and future bioclimatic niches for *M. celadussa* and *Z. filipendulae* in Switzerland, we calibrated our models with the occurrences of species obtained and with 100,000 randomly (0.08% of the raster cells) selected background points across the Swiss territory for *Z. filipendulae* and in western Switzerland for *M. celadussa*. For *M. celadussa*, we only keep observations made in western Switzerland, as this species is included in an aggregate containing another species (*M. athalia*) which is distributed in eastern Switzerland. Data for eastern Switzerland were thus missing, and to avoid creating biases in our model, we excluded the regions colonized by *M. athalia* in both models and maps (Figure S2). To characterise current (1981 to 2010) and future (2085, i.e., average 2075 to 2095) climatic niches, we used 4 bioclimatic variables (Karger et al., 2017; <https://chelsa-climate.org/>); (i) bioclim2: mean diurnal air temperature range (mean of monthly [max. temperature - min. temperature]), (ii) bioclim5: mean daily air temperature of the warmest month, (iii) bioclim12: annual precipitation amount, and (iv) bioclim15: precipitation seasonality. These four climatic variables were selected as they are known to be important predictors for species occurrence (Rusconi et al., 2022) and had a low pair-wise correlation (computation of matrix of Pearson and Spearman rank-correlation <0.73). For future climatic scenarios (2085), we kept the same bioclimatic variables and selected a Global Circulation Model (GCM) HadGEM2-AO (Collins et al., 2008) with two Representative Concentration Pathways (RCP): RCP 4.5 and RCP 8.5 (IPCC, 2014; Van Vuuren et al., 2011). The raster used for the bioclimatic variables were from the CHclim25 dataset (CH2018 Project Team, 2018) and had a resolution of 25 x 25 m. To build species niche distribution maps, we used a maximum entropy model (Elith et al., 2011; Phillips et al., 2006) in R using the packages “enmSdmX” package (Smith et al., 2023), “terra” (Hijmans, 2023) and “dismo” (Hijmans et al., 2023). The model was trained 30 times and elevation data were obtained with the digital height model of Switzerland (DHM25, Federal Office of Topography swisstopo) which has a precision of 25 m. Plots were made using “ggplot2” (Wickham, 2016). Finally, we evaluate our models by split resampling with a calibration on 80% and an evaluation on 20% of the data (Descombes et al., 2020). Our models have reliable predictions, with AUC values > 0.7.

## Estimating the migration rate of M. celadussa and Z. filipendulae

To calculate average migration rates per decade for *M. celadussa* and *Z. filipendulae,* we used similar methods as Vitasse et al. (2021), using data obtained between 1985 and 2022. Although some occurrence data are older than 1985, the number of observations is scarce and where thus excluded to avoid biases. Based on observations scored with a precision of at least 1 km^2^ we calculated the median and the 95^th^ percentile of elevational distributions for each year from 1985 to 2022. Duplicated data were excluded and missing elevation data were estimated using the digital height model of Switzerland (DHM25, Federal Office of Topography swisstopo).

We fitted separate linear models for *M. celadussa* or *Z. filipendulae*, for which the median or the 95^th^ quantile of the elevation distribution were used as response variables and the time (year of the records) as the explanatory variable. Effects and confidence intervals of time (years) on the elevation distribution were estimated by performing F-tests tests of fitted models using ANOVA. Models were evaluated using

the packages DHARMa (Hartig, 2022) and Stats (R Core Team, 2021).

# Supplementary results

**Table S1. Information on focal plant species.** Information reported in this table is based on Lauber *et al.* (2018), Rønsted *et al.* (2000), Zagrobelny *et al.* (2007).

|  | ***Plantago lanceolata*** | ***Plantago atrata*** | ***Lotus corniculatus*** | ***Lotus alpinus*** |
| --- | --- | --- | --- | --- |
| **Pollination** | Wind | Wind | Insects | Insects |
| **Chemical defences** | Iridoid glycosides | Iridoid glycosides | Cyanogenic glycosides | Cyanogenic glycosides |
| **Seed dispersion** | Animals | Animals | Animals | Animals |
| **Focal specialist butterfly** | *Melitaea celadussa* | *Melitaea celadussa* | *Zygaena filipendulae* | *Zygaena filipendulae* |
| **Elevational distribution** | Collinear-montane (-subalpine) | Subalpine-alpine | Collinear-montane | Subalpine-alpine |

**Table S2: Summary of sample sizes and caterpillar instars used for each experiment.** The oviposition experiment was performed on the field at low (1500 m) and high (2150 m) elevation. Experiments under controlled conditions exploring responses to host plant shifts/translocation effects reflected in caterpillar growth were performed using plants included in the field experiment, growing at either high or low elevations. Experiments testing responses to host plant shifts reflected in caterpillar growth, preference, diapause/pupation rates and wing area were performed under controlled conditions using plants collected at low and high elevation sites. For these experiments, high-elevation plants were collected around the experimental site at 2150 m, while low-elevation plants were collected in the vicinity of the greenhouses where controlled experiments were performed. Diapause/pupation rates and wing area were measured only for *M. celadussa* as all caterpillars of *Z. filipendulae* went to diapause. Samples sizes for “Chemical analyses” refer to the number of plants used for the assessment of IGs and CNGs in *Plantago* spp. and *Lotus* spp., respectively. Numbers in parentheses for the oviposition experiment indicate the original replication of the experiment when set up in the field for the oviposition experiment and the initial number of replicates for the caterpillar growth while bold numbers indicated for both the final sample size.

|  | | ***M. celadussa*** | | ***Z. filipendulae*** | | **Stages / instars** |
| --- | --- | --- | --- | --- | --- | --- |
|  |  | *P. lanceolata* | *P. atrata* | *L. corniculatus* | *L. alpinus* |  |
| **Oviposition** | *1500 m* | **10** (11) | | **5** (15) | | Adults |
|  | *2150 m* | **6** (9) | | **5** (15) | |  |
| **Caterpillar growth** | *1500 m* | **35** (43) | **17** (36) | **18** (27) | **20** (30) | L2-L3 |
|  | *2150 m* | **34** (42) | **42** (46) | **25** (30) | **14** (20) |  |
| **Caterpillar preference** | | **39** | | **40** | | L3-L6 |
| **Diapause and pupation** | | **30** | **27** | All caterpillars entered diapause | | L2-Pupae/diapause |
| **Wing area** | | **17** | **11** |  |  | Adults |
| **Chemical analyses** | *1500 m* | **8** | **8** | **6** | **8** | NA |
|  | *2150 m* | **7** | **7** | **7** | **8** | NA |

**Table S3. Information on experimental sites.** The study sites are located in the Western Swiss Alps, in the Municipality of Lavey-Morcles, on the West side of the Mt. Dents de Morcles. Temperature was recorded with one HOBO datalogger (Model UA-002-64, Onset, Bourne, USA) placed c. 5 cm above ground at each site during the experiment (28^th^ of June - 31^st^ of July 2022).

|  | **Low-elevation site** | **High-elevation site** |
| --- | --- | --- |
| **Elevation** | 1505 m | 2157 m |
| **GPS coordinates** | 46°12′58″N, 7°02′47″E | 46°12′13″N, 7°03′32″E |
| **Meadow type** | Cow pasture | Sheep pasture |
| **Average temperature** | 21.0 °C | 18.2 °C |

**Table S4. Information on sites at which plants were harvested and Lepidoptera species captured.** Plant and Lepidoptera individuals included in this study originated from multiple locations across the Western Swiss Alps (Canton of Vaud). *M. celadussa* and *Z. filipendulae* were captured in dry meadows in the municipality of Lavey-Morcles and Ollon. Note that high-elevation plants included in the study were collected in the surroundings of the experimental field site situated at high-elevation (Rionda; 2150 m), while low-elevation plants included in caterpillar preference, pupation and wing size experiments were collected from sites situated at 500 m, close to the greenhouses where controlled experiments were performed (University of Neuchâtel, Switzerland).

| **Site** | **GPS coordinates** | **Elevation (m)** | **Species and number of individuals** |
| --- | --- | --- | --- |
| Rosseline | 7°02'26.084" E  46°12’57.979” N | 1325-1510 | *P. lanceolata* (n = 20),  *L. corniculatus* (n = 30) |
| Rionda | 7°03'39.823" E  46°12’19.554” N | 2090-2220 | *P. atrata* (n = 20),  *L. alpinus* (n = 30) |
| University of Neuchâtel | 6°57'00.114" E  47°00’01.204” N | 485-500 | *P. lanceolata* (n = 39),  *L. corniculatus* (n = 29) |
| Lavey-Morcles | 7°01′55.858″ E  46°12′00.685″ N | 755-920 | *M. celadussa* (n = 9)*,*  *Z. filipendulae* (n = 24) |
| Lavey | 7°01'32.689" E  46°11’52.273” N | 420-440 | *Z. filipendulae* (n = 9) |
| Morcles | 7°02'3.342" E  46°12’10.506” N | 1020-1105 | *M. celadussa* (n = 3)*,*  *Z. filipendulae* (n = 30) |
| Antagnes 1 | 7°00'42.457" E  46°17’2.383” N | 675-705 | *M. celadussa* (n = 2) |
| Antagnes 2 | 7°00'50.727" E  46°16’53.143” N | 670 | *M. celadussa* (n = 1) |
| Antagnes 3 | 7°01'5.039" E  46°16'36.143" N | 610-630 | *M. celadussa* (n = 45)*,*  *Z. filipendulae* (n = 15) |
| Col de la Croix | 7°05'7.161" E  46°18’10.945” N | 1395-1460 | *Z. filipendulae* (n = 6) |
| Chesières | 7°02'1.748" E  46°17'51.596" N | 1180-1225 | *Z. filipendulae* (n = 3) |
| Huémoz | 7°01'8.012" E  46°17'43.738" N | 990-1090 | *Z. filipendulae* (n = 3) |

**Table S5. Effects of host plant identity and elevation on morphological traits for *Plantago* spp. and *Lotus* spp.** The table shows results from PERMANOVA analyses using Bray-Curtis based on a PCA analysing divergence in morphological traits reflected captured by stalk height, leaf number and inflorescence number between low- (*P. lanceolata* and *L. corniculatus*) and high-elevation host plants (*P. atrata* and *L.* alpinus) for each genus separately. Effects of host plant identity and elevation were estimated by F-tests. Significant effects are indicated in bold (p-value).

| **Species group** | **Term** | **F-value** | **df** | **p-value** |
| --- | --- | --- | --- | --- |
| ***Plantago* spp.** | Host plant identity | 20.19 | 1 | **<0.001** |
|  | Elevation | 2.35 | 1 | 0.104 |
|  | Host plant identity $\times$ Elevation | 2.69 | 1 | 0.077 |
| ***Lotus* spp.** | Host plant identity | 15.58 | 1 | **<0.001** |
|  | Elevation | 0.47 | 1 | 0.689 |
|  | Host plant identity $\times$ Elevation | 1.88 | 1 | 0.145 |

**Table S6. Effects of host plant identity and elevation on C:N ratios for *Plantago* spp. and *Lotus* spp.** Effects of host plant identity across species (*P. lanceolata*, *P. atrata*, *L. corniculatus* and *L.* alpinus) and elevation were estimated by F-tests. Significant effects are indicated in bold (p-value).

| **Term** | **F-value** | **df** | **p-value** |
| --- | --- | --- | --- |
| Host plant identity | 4.23 | 3 | **0.010** |
| Elevation | 0.96 | 1 | 0.333 |
| Host plant identity $\times$ Elevation | 0.72 | 3 | 0.543 |

**Table S7. Effects of host plant identity and elevation on chemical defence compounds for *M. celadussa* and *Z. filipendulae*.** Effects of host plant identity and elevation were estimated by F-tests. Significant effects are indicated in bold (p-value).

| **Compound** | **Term** | **F-value** | **df** | **p-value** |
| --- | --- | --- | --- | --- |
| **Catapol** | Host plant identity | 4.42 | 1 | **0.046** |
|  | Elevation | 1.33 | 1 | 0.259 |
|  | Host plant identity $\times$ Elevation | 0.77 | 1 | 0.388 |
|  | Residuals |  | 24 |  |
| **Aucubin** | Host plant identity | 0.45 | 1 | 0.508 |
|  | Elevation | 0.11 | 1 | 0.739 |
|  | Host plant identity $\times$ Elevation | 0.07 | 1 | 0.792 |
|  | Residuals |  | 24 |  |
| **Linamarin** | Host plant identity | 31.03 | 1 | **<0.001** |
|  | Elevation | 0.39 | 1 | 0.538 |
|  | Host plant identity $\times$ Elevation | 2.67 | 1 | 0.114 |
|  | Residuals |  | 27 |  |
| **Lotaustralin** | Host plant identity | 15.49 | 1 | **<0.001** |
|  | Elevation | 2.48 | 1 | 0.127 |
|  | Host plant identity $\times$ Elevation | 0.41 | 1 | 0.528 |
|  | Residuals |  | 27 |  |

**Figures**


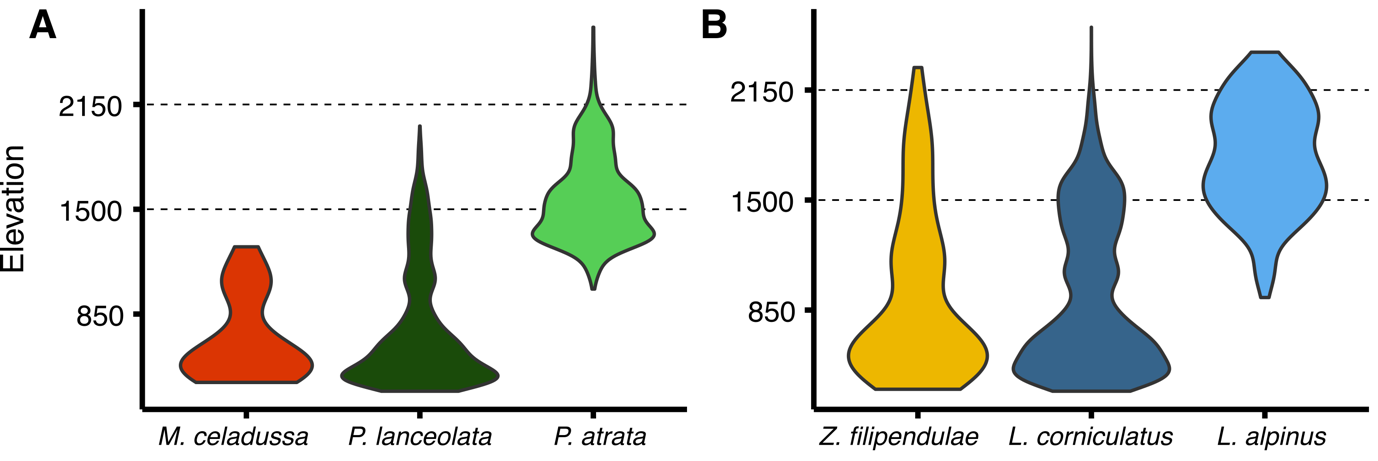


**Figure S1. Elevational distribution of *M. celadussa* (a) and *Z. filipendulae* (B) and their current low- and novel high-elevation host plants in the cantons of Vaud and Valis.** (a) shows the elevational distribution (y-axis) of *M. celadussa* and its current low- and potential novel high-elevation host plants, *P. lanceolata* and *P. atrata*, respectively. (b) shows the elevational distribution (y-axis) of *Z. filipendulae* and its current low- and potential novel high-elevation host plants, *L. corniculatus* and *L. alpinus*, respectively. Dashed horizontal lines indicate the elevation of experimental sites. Data on *M. celadussa* and *Z. filipendulae* were provided by info fauna (2023), while data on the plants were provided by Info Flora (2023). These graphics are based on observations made in the region in which the field experiment was performed, and Lepidopterans/plant species were collected (cantons of Vaud and Valais, Switzerland). Note that high-elevation plants were not observed below 1750 m in the elevation gradient in focus for this study.


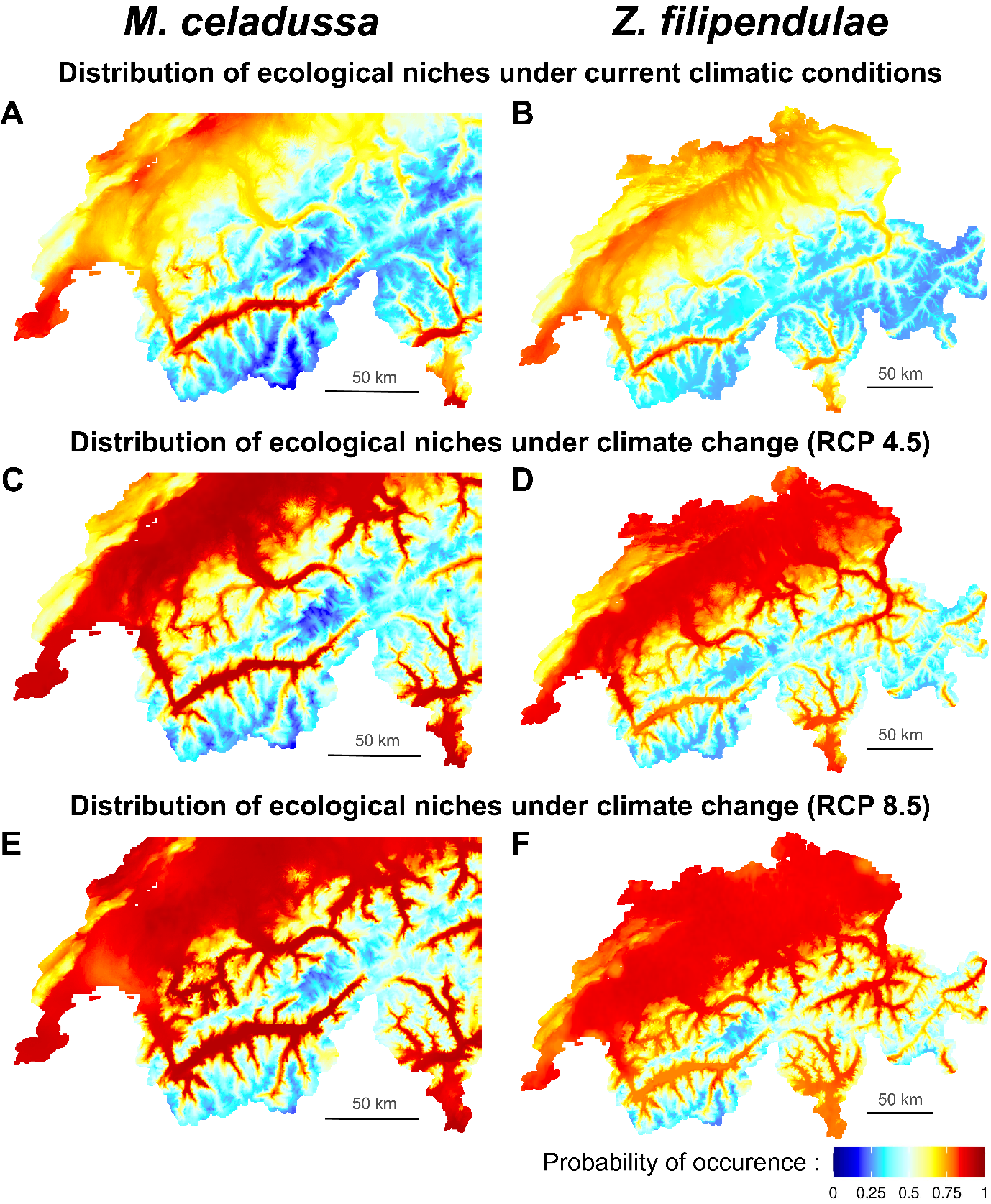


**Figure S2. Illustrations of the modelized climatic niches of *M. celadussa* (a, c, e) and *Z. filipendulae* (b, d, f) under current climatic conditions and future (RCP 4.5 and RCP 8.5) climatic scenarios.** (a) shows the probability of occurrence of *M. celadussa* in Western Switzerland, while (b) shows the probability of occurrence of *Z. filipendulae* across Switzerland under current climatic conditions. (c) and (d) show predictions of the probability of occurrence for *M. celadussa* and *Z. filipendulae*, respectively, for 2085 under RCP 4.5 climate change scenario). (e) and (f) show predictions of the probability of occurrence for *M. celadussa* and *Z. filipendulae*, respectively, for 2085 under RCP 8.5 climate change scenario. Maps are based on data obtained from species distribution models based on the occurrence data of the National Data and Information Center on the Swiss Fauna and bioclimatic data from the CHclim25 dataset (CH2018 Project Team, 2018). The resolution of maps is at a scale of 25 x 25 m.


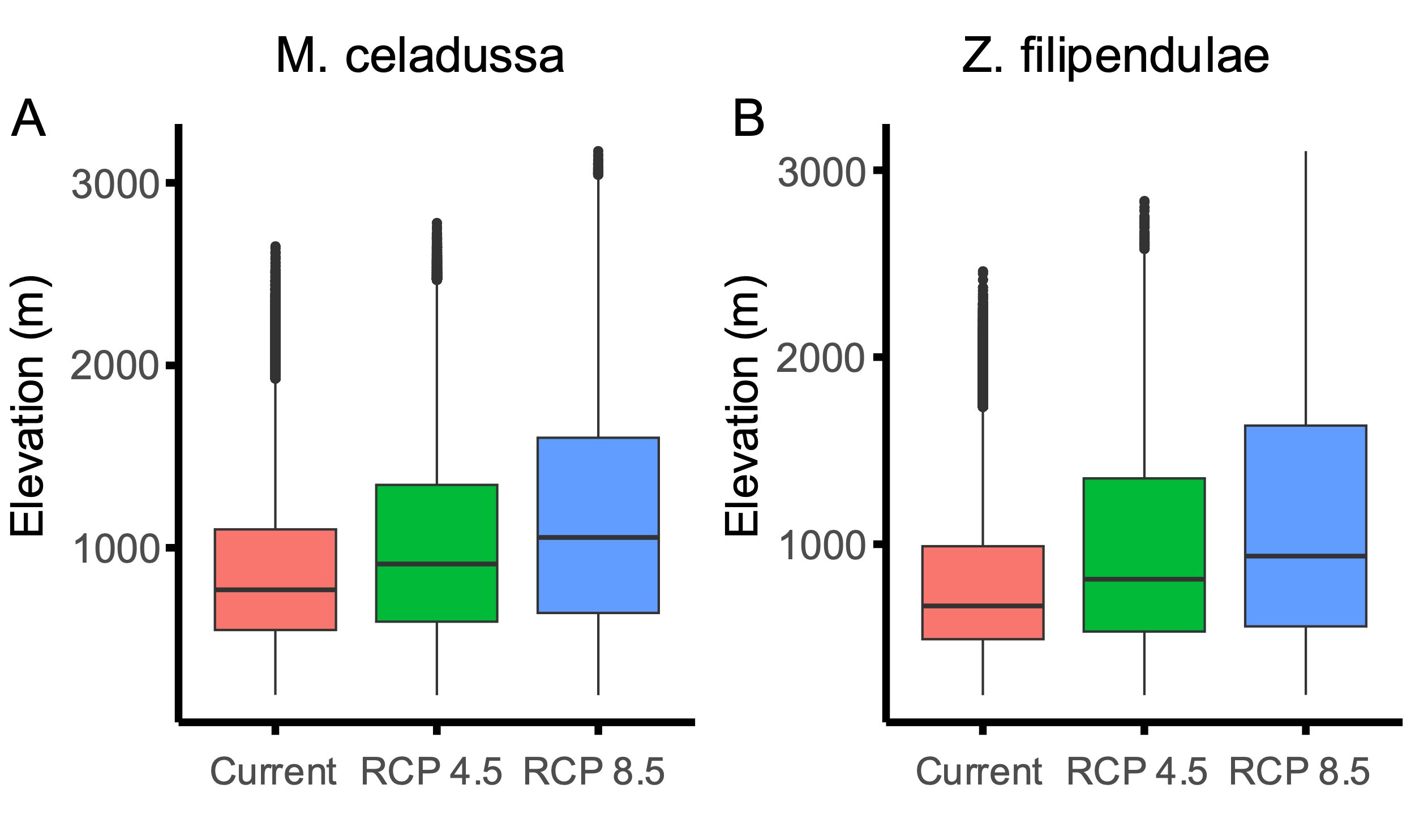


**Figure S3. Shift in elevation of the climatic niches of *M. celadussa* (a) and *Z. filipendulae* (b).** (a) and (b) show the elevation distribution (y-axis) of suitable environments (probability of occurrence > 0.5) for *M. celdussa* *Z. filipendulae*, respectively, according to current and predicted future (2085; RCP 4.5 and 8.5) climates (x-axis). Elevation data were obtained from our spatial distribution models and their projection under different climate scenarios with the digital height model of Switzerland (DHM25, Federal Office of Topography swisstopo) which has a precision of 25 m. Bold, horizontal lines show medians, while vertical bars illustrate distributions of the 1st - 4th quantiles and points represent outliers.


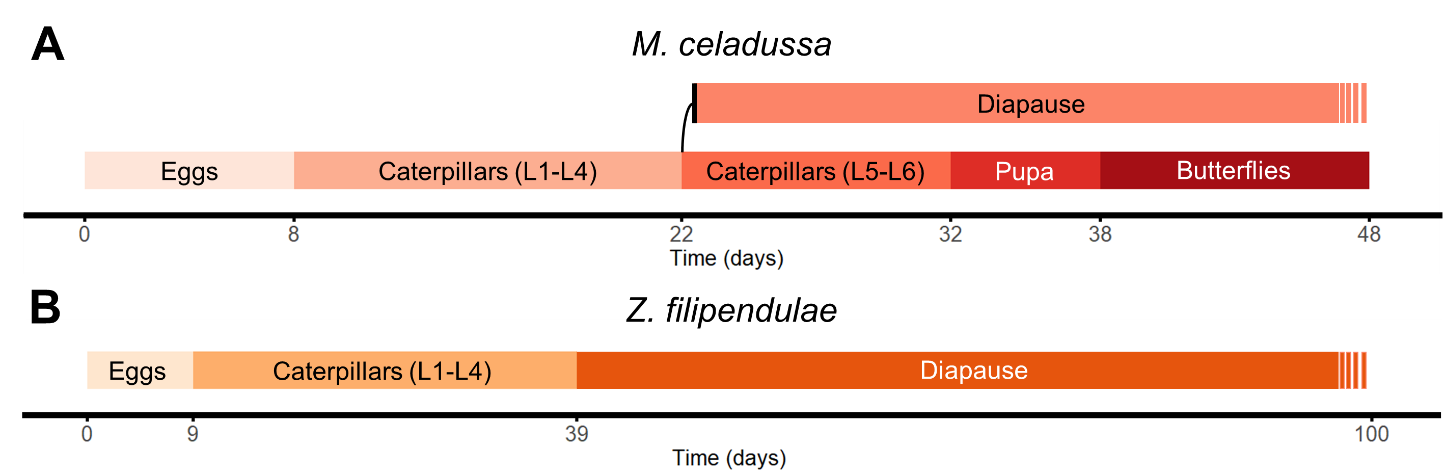


**Figure S4. Life cycles of *M. celadussa* (a) and *Z. filipendulae* (b) observed under controlled conditions performed in this study.** (a) and (b) show the life cycle of *M. celadussa* and *Z. filipendulae*, respectively, with the duration of life stages in days (x-axis). Life stages are indicated by colour. Data were collected during 4 generations for *M. celadussa* which was reared for six months. All *Z. filipendulae* caterpillars entered diapause at the fourth instar (b), thus multiple generations could not be obtained for this species. “L” followed by a number refers to the instar of the caterpillars.

**
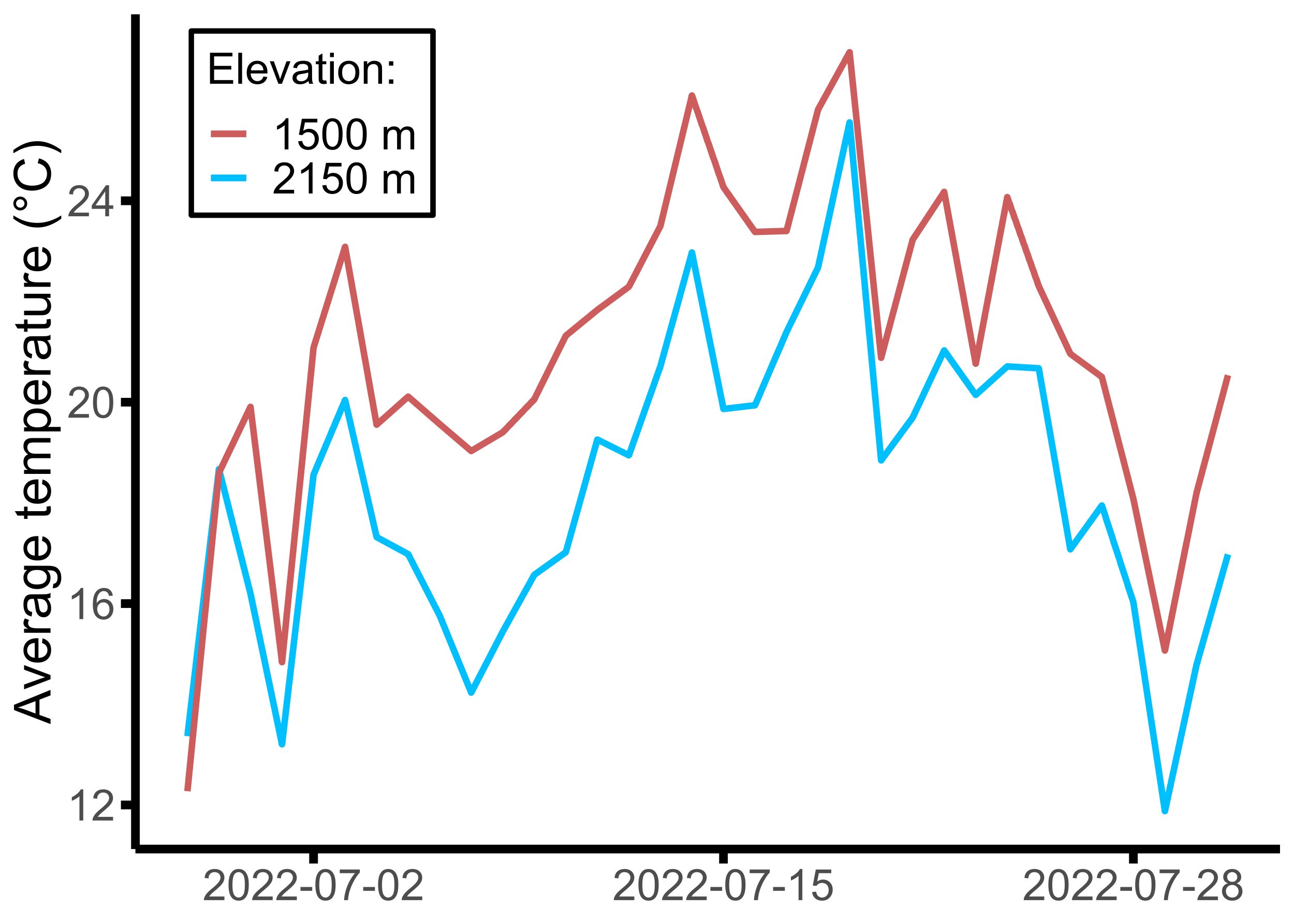
**

**Figure S5. Daily mean temperature at experimental sites situated at 1500 and 2150 m.** Above-ground temperatures were recorded with one HOBO datalogger (Model UA-002-64, Onset, Bourne, USA) placed c. 5 cm above ground at each site during the experiment (28^th^ of June - 31^th^ of July 2022). Blue line shows the temperature at the high-elevation site (2150 m) while the red line illustrates the temperature at the low-elevation site (1500 m).

**
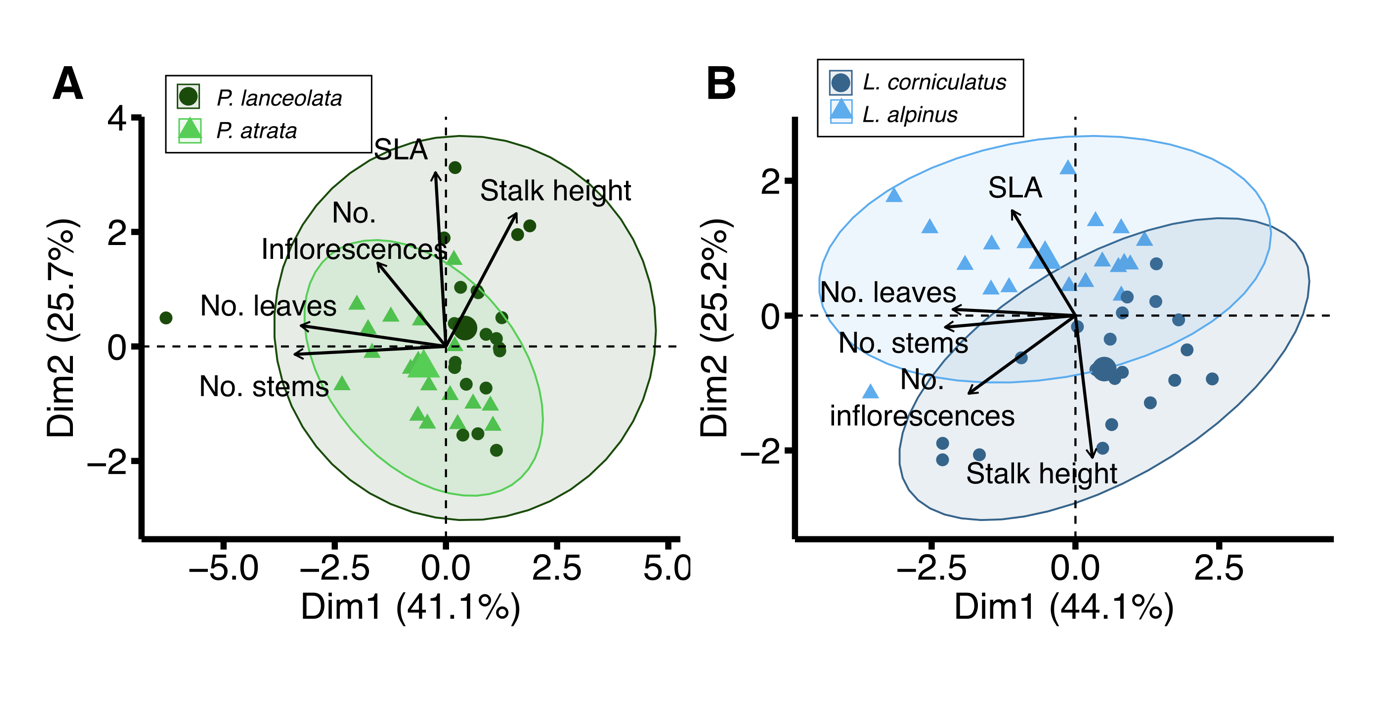
**

**Figure S6. Divergence of plant traits across low- and high-elevation host plants (a-b).** (a) and (b) show the first (x-axis) and second (y-axis) component axes from Principal Component Analysis (PCA), illustrating divergence of plant traits based on estimates of SLA, stalk height, number of inflorescences, number of leaves and number of stems for *Plantago* spp. (*P. lanceolata* and *P. atrata*) and *Lotus* spp. (*L. corniculatus* and *L. alpinus*), respectively. Ellipses represent clustering of 95% CI of elevations, assigned by colour (dark and light green/blue for low- and high-elevation host plants, respectively). Each individual is represented by points for which colour and shape indicate species identity. Enlarged points indicate means for each species. Note that effects of elevation are not illustrated here, as they were not significant. For details on significant effects of host plant identity and elevation on the divergence of morphological traits, see Table S4.

**
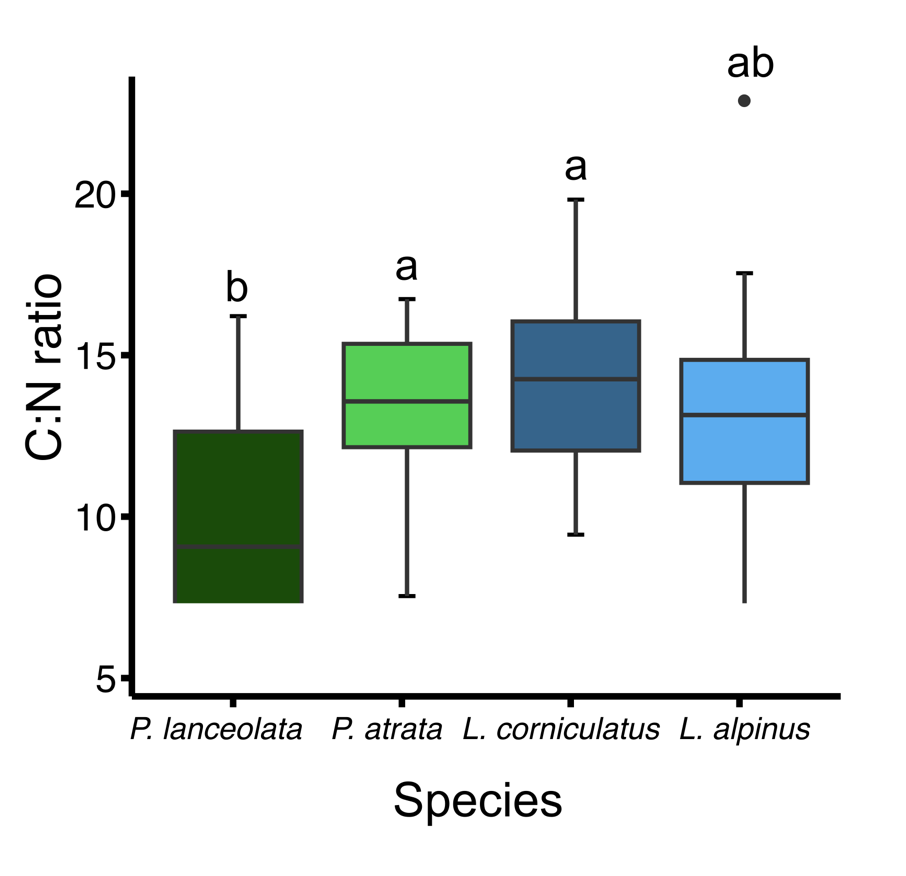
**

**Figure S7. Carbon and Nitrogen (C:N) ratios in leaves for low- and high-elevation host plants.** This figure shows C:N ratios (carbon concentration divided by nitrogen concentration) on the y-axis for each host plant identity (x-axis). *P. lanceolata* and *P. atrata* are illustrated in dark and light green, respectively, and *L. corniculatus* and *L. alpinus* in dark and light blue, respectively. Bold, horizontal lines show medians, error bars illustrate distributions of the 1st - 4th quantile and points represent outliers. Letters indicate significant differences between host plant identities (p < 0.05) calculated with F-tests. Note that effects of elevation are not illustrated here, as elevation had no impact on C:N ratios. For details on effects of host plant identity and elevation on C:N ratios, see Table S5.

**
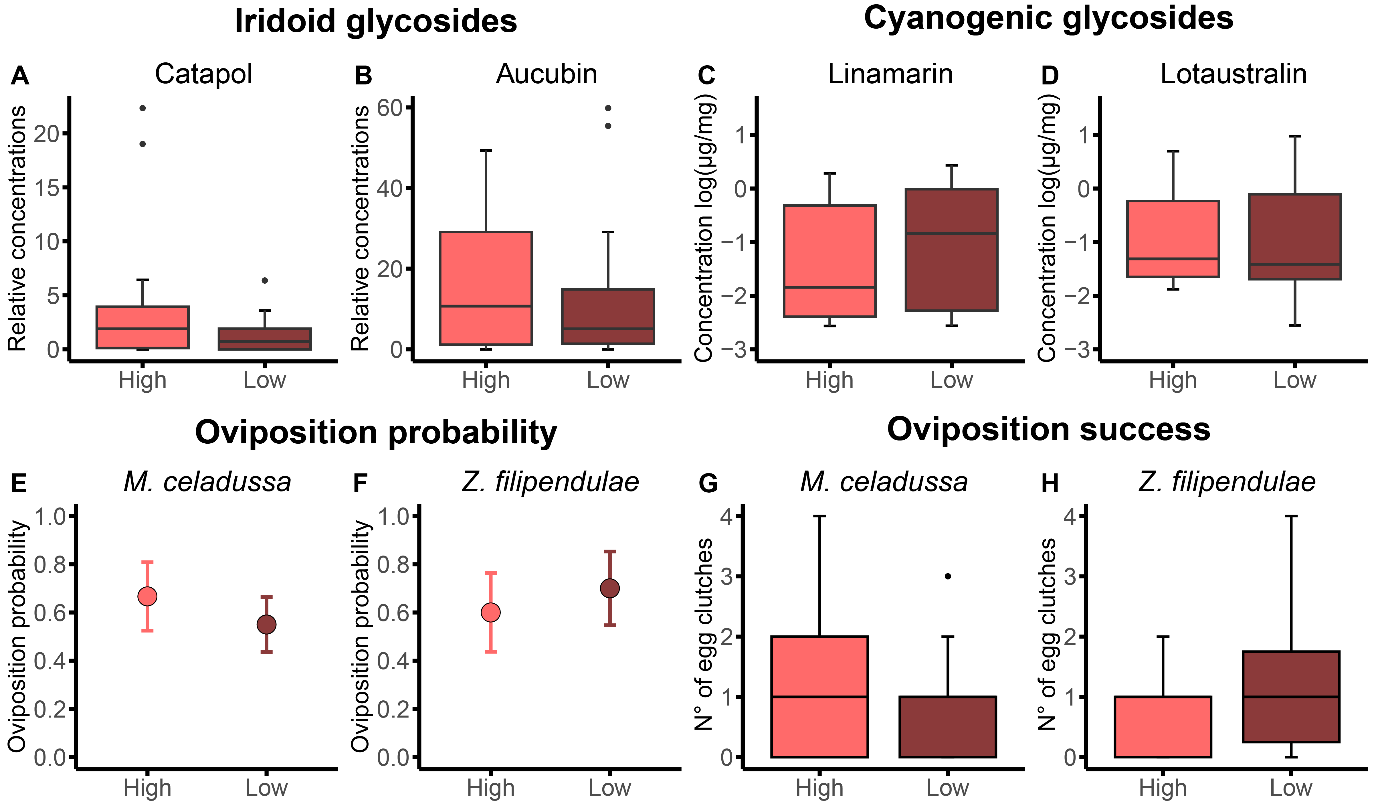
**

**Figure S8. Effects of elevation on relative concentrations of catapol and aucubin (a-b) for *P. lanceolata* and *P. atrata*, absolute concentrations of linamarin and lotaustralin (c-d) in *L. corniculatus* and *L. alpinus*, oviposition probability (e-f) and caterpillar preference (g-h).** (a-b) illustrate relative concentrations of catapol and aucubin on the y-axis across elevations (x-axis). (c-d) illustrate absolute concentrations log(µg/mg) of linamarin and lotaustralin on the y-axis across elevations (x-axis). (e-f) show mean oviposition probability on the y-axis for each elevation (x-axis). (g-h) show the number of egg clutches (y-axis) for each elevation (x-axis). For boxplots (a-d, g-h), bold, horizontal lines show medians, error bars illustrate distributions of the 1^st^ - 4^th^ quantile and points represent outliers. For (e-f), points indicate means and error bars illustrate ± 1 standard error.

**REFERENCES**

Bakhtiari, M., Formenti, L., Caggìa, V., Glauser, G., & Rasmann, S. (2019). Variable effects on growth and defense traits for plant ecotypic differentiation and phenotypic plasticity along elevation gradients. *Ecology and Evolution*, *9*(7), 3740-3755. https://doi.org/10.1002/ece3.4999

CH2018 Project Team (2018). CH2018 - Climate Scenarios for Switzerland. National Centre for Climate Services. https://doi.org/10.18751/Climate/Scenarios/CH2018/1.0

Collins, W., Bellouin, N., Doutriaux-Boucher, M., Gedney, N., Hinton, T., Jones, C., Liddicoat, S., Martin, G., O’Connor, F., & Rae, J. (2008). Evaluation of the HadGEM2 model. Met Office Exeter, UK.

Descombes, P., Pitteloud, C., Glauser, G., Defossez, E., Kergunteuil, A., Allard, P.-M., Rasmann, S., & Pellissier, L. (2020). Novel trophic interactions under climate change promote alpine plant coexistence. *Science*, *370*(6523), 1469-1473. https://doi.org/10.1126/science.abd701

Elith, J., Phillips, S. J., Hastie, T., Dudík, M., Chee, Y. E., & Yates, C. J. (2011). A statistical explanation of MaxEnt for ecologists. *Diversity and distributions*, *17*(1), 43-57. https://doi.org/10.1111/j.1472-4642.2010.00725.x

Formenti, L., Caggìa, V., Puissant, J., Goodall, T., Glauser, G., Griffiths, R., & Rasmann, S. (2021). The effect of root‐associated microbes on plant growth and chemical defence traits across two contrasted elevations. *Journal of Ecology*, *109*(1), 38-50. https://doi.org/10.1111/1365-2745.13440

Hartig, F. (2022). DHARMa: Residual Diagnostics for Hierarchical (Multi-Level / Mixed) Regression Models. In. https://CRAN.R-project.org/package=DHARMa

Hijmans, R. J. (2023). terra: Spatial Data Analysis. In https://CRAN.R-project.org/package=terra

Hijmans, R. J., Phillips, S., Leathwick, J., & Elith, J. (2023). dismo: Species Distribution Modeling. https://CRAN.R-project.org/package=dismo

IPCC. (2014). Climate change 2014: synthesis report. Contribution of Working Groups I, II and III to the fifth assessment report of the Intergovernmental Panel on Climate Change. IPCC: Geneva, Szwitzerland.

Karger, D. N., Conrad, O., Böhner, J., Kawohl, T., Kreft, H., Soria-Auza, R. W., Zimmermann, N. E., Linder, H. P., & Kessler, M. (2017). Climatologies at high resolution for the earth’s land surface areas. *Scientific data*, *4*(1), 1-20. https://doi.org/10.1038/sdata.2017.122

Kassambara, A., & Mundt, F. (2020). factoextra: Extract and Visualize the Results of Multivariate Data Analyses. In. https://CRAN.R-project.org/package=factoextra

Lauber, K., Wagner, G., & Gygax, A. (2018). *Flora Helvetica* (Haupt ed.).

Lenth, R. V. (2022). emmeans: Estimated Marginal Means, aka Least-Squares Means. In. https://CRAN.R-project.org/package=emmeans

Litman, J., Chittaro, Y., Birrer, S., Praz, C., Wermeille, E., Fluri, M., Stalling, T., Schmid, S., Wyler, S., & Gonseth, Y. (2018). A DNA barcode reference library for Swiss butterflies and forester moths as a tool for species identification, systematics and conservation. *PLoS One*, *13*(12), e0208639. https://doi.org/10.1371/journal.pone.0208639

Oksanen, J., Blanchet, F. G., Friendly, M., Kindt, R., Legendre, P., McGlinn, D., Minchin, P. R., O'Hara, R. B., Simpson, G. L., Solymos, P., Stevens, M. H. H., Szoecs, E., & Wagner, H. (2020). vegan: Community Ecology Package. In. https://CRAN.R-project.org/package=vegan

Phillips, S. J., Anderson, R. P., & Schapire, R. E. (2006). Maximum entropy modeling of species geographic distributions. *Ecological Modelling*, *190*(3-4), 231-259. https://doi.org/10.1016/j.ecolmodel.2005.03.026

R Core Team. (2021). R: A Language and Environment for Statistical Computing. https://www.r-project.org/

Rønsted, N., Göbel, E., Franzyk, H., Jensen, S. R., & Olsen, C. E. (2000). Chemotaxonomy of Plantago. Iridoid glucosides and caffeoyl phenylethanoid glycosides. *Phytochemistry*, *55*(4), 337-348. https://doi.org/10.1016/S0031-9422(00)00306-X

Rusconi, O., Broennimann, O., Storrer, Y., Le Bayon, R. C., Guisan, A., & Rasmann, S. (2022). Detecting preservation and reintroduction sites for endangered plant species using a two‐step modeling and field approach. *Conservation Science and Practice*, *4*(10), e12800. https://doi.org/10.1111/csp2.12800

Shlichta, J. G., Glauser, G., & Benrey, B. (2014). Variation in Cyanogenic Glycosides Across Populations of Wild Lima Beans (Phaseolus lunatus) Has No Apparent Effect on Bruchid Beetle Performance. *Journal of Chemical Ecology*, *40*(5), 468-475. https://doi.org/10.1007/s10886-014-0434-0

Schneider, C. A., Rasband, W. S., & Eliceiri, K. W. (2012). NIH Image to ImageJ: 25 years of image analysis. *Nature methods*, *9*(7), 671-675. https://doi.org/10.1038/nmeth.2089

Smith, A. B., Murphy S. J., Henderson D., & L., E. K. (2023). Including imprecisely georeferenced specimens improves accuracy of species distribution models and estimates of niche breadth. *Global Ecology & Biogeography*, *32*, 1-13. https://doi.org/10.1111/geb.13628

Van Vuuren, D. P., Edmonds, J., Kainuma, M., Riahi, K., Thomson, A., Hibbard, K., Hurtt, G. C., Kram, T., Krey, V., & Lamarque, J.-F. (2011). The representative concentration pathways: an overview. *Climatic Change*, *109*, 5-31. https://doi.org/10.1007/s10584-011-0148-z

Vitasse, Y., Ursenbacher, S., Klein, G., Bohnenstengel, T., Chittaro, Y., Delestrade, A., Monnerat, C., Rebetez, M., Rixen, C., Strebel, N., Schmidt, B. R., Wipf, S., Wohlgemuth, T., Yoccoz, N. G., & Lenoir, J. (2021). Phenological and elevational shifts of plants, animals and fungi under climate change in the European Alps. *Biological Reviews*, *96*(5), 1816-1835. https://doi.org/10.1111/brv.12727

Wickham, H. (2016). ggplot2: Elegant Graphics for Data Analysis. Springer-Verlag New York. https://ggplot2.tidyverse.org

Zagrobelny, M., Bak, S., Thorn Ekstrøm, C., Erik Olsen, C., & Lindberg Møller, B. (2007). The cyanogenic glucoside composition of Zygaena filipendulae (Lepidoptera: Zygaenidae) as effected by feeding on wild-type and transgenic lotus populations with variable cyanogenic glucoside profiles. *Insect Biochemistry and Molecular Biology*, *37*(1), 10-18. https://doi.org/10.1016/j.ibmb.2006.09.008
